# Hierarchically Ordered *α*-Zirconium Phosphate Platelets in Aqueous Phase with Empty Liquid

**DOI:** 10.1038/s41598-019-51934-y

**Published:** 2019-11-08

**Authors:** Xi Lin, Dirk Schmelter, Sadaf Imanian, Horst Hintze-Bruening

**Affiliations:** BASF’s Coatings Division, 48165 Muenster, Germany

**Keywords:** Materials science, Nanoscale materials, Soft materials

## Abstract

Platelets of *α*-zirconium phosphate (*α*-ZrP) obtained from the reflux method in H_3_PO_4_ are successfully exfoliated into water via the intercalation of alkanol amines. With volume fractions greater than 0.02 they are stacked into tactoids of few layers with a repeat distance in the order of 10 nm. The tactoids align into nematic liquid crystalline phases with irregularly wide interstices of empty liquid. Colloidal processing involves the freeze-drying of such anisotropic fluids and the dispersion of the restacked tacoids into aqueous dispersions of colloidal polymer particles of largely varying size which occupy the otherwise empty liquid between the *α*-ZrP tactoids and induce piling of the tactoids into columns. Real-time SAXS on drying films and TEM of the obtained coatings demonstrate that the stacked *α*-ZrP platelets and the polymer particles comprising liquid dry separately without polymer intercalation, while the morphology of the obtained composites can be tuned primarily by the size of the polymer colloids. Concomitant *α*-ZrP hydrolysis in the exfoliation step is scrutinized as a function of amine basicity and temperature. The role of zirconium based hydrolysis products in the hierarchical *α*-ZrP assembly is indirectly though consistently confirmed by opposing impacts of ultra-filtration and added oxoanions on the platelets’ spacing, smoothness and aggregation. HAADF-TEM imaging of scattered, singular platelets and XRD peak analysis of the pristine solid shed light on the *α*-ZrP synthesis. Coexisting flakes and lacunae, both similar in size to the intra-layer crystal domains, suggest the stitching of proto-*α*-ZrP flakes into extended layers in accordance with our observations on the aging behaviour of *α*-ZrP dispersions as well as with literature data on related systems.

## Introduction

**Figure 1:**
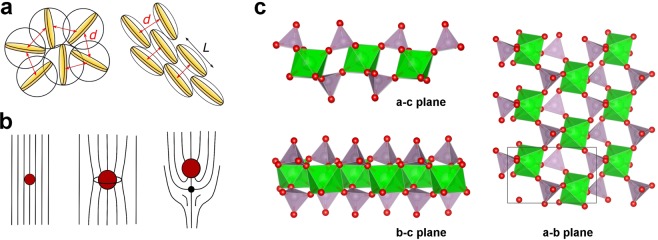
(**a**) Isotropic to nematic phase transition of 2D particles with lateral size *L*. (**b**) Topological point defects (radial and hyperbolic “hedgehog”) in a nematic fluid caused by spheres above a critical size *R*_*c*_ ≈ *K*/*W* [m] known as de Gennes-Kléman length with *K* [N] the elasticity constant of the director field and *W* [J/m^2^] the surface anchoring coefficient for homeotropic conditions^13^. (**c**) Section of a single *α*-ZrP layer (Zr(HPO_4_)_2_ · H_2_O) projected along the unit cell axes. Zirconium octahedra (green) are linked to phosphate tetrahedra (grey), the hydrogens of their apical P-OH groups being omitted.

Over the last decade the concept of empty liquids has been discussed in the context of the directional assembly of patchy colloids^1–3^. Nevertheless, Ruzicka *et al*. have shown that empty liquid is also the corollary of the assembly of patchy 2D particles (Laponite RD) into clusters in aqueous phase^4^. In volume fractions sufficient for the percolation of such clusters, these are commonly used to render aqueous preparations like cosmetics or paints shear-thinning, often associated with distinct yield points. Contrary to these tiny, randomly oriented hectorite platelets, homogeneous alignment of 2D particles with large aspect ratios has been shown to provide a barrier function^5–7^ and mechanical reinforcement^8^ to polymer films as well as to enhance effect pigment orientation in organic coatings^9^. These properties lead to better substrate protection against corrosion and stone impact, respectively, whereas metallic effect coatings display larger viewing angle dependent reflectivity. In the pristine liquid paint or during its drying such 2D particles undergo an isotropic to nematic (I-N) phase transition above a threshold volume fraction which is driven by entropy gain through a reduced excluded volume (Fig. 1a)^10^. Admittedly, a dense packing in the lyotropic liquid crystalline phase with an increased number of nearest neighbours^11,12^ poses an obstacle for the formulation of organic coatings, which usually involves colloidal polymer particles whose diameters may surpass the interlayer distances of the 2D nanoparticles. Consequently, the presence of such large objects disturbs the liquid crystalline order through topological point defects (Fig. 1b)^13^. We note that this effect can as well be used creatively to arrange nano-particles in thermotropic nematics^14^. Nevertheless, such applications imply inverse volume fractions of the 2D and 3D particles.

Accordingly, the stabilization of nano-platelets with polymer brushes and their exfoliation into solvent-borne, lyotropic smectic phases^15–17^ has been used for the formulation of organic coatings via the addition of molecularly dissolved monomers and polymers^17–21^. Such coatings have been proposed for anti-corrosive^6,22^ and anti-scratch^23^ applications. However, the polymer quantity that is needed to obtain a sufficiently dense and thick brush layer for steric stabilization narrows its applicability. With regard to the cited *α*-ZrP platelets (*α*-zirconium phosphate, Zr(HPO_4_)_2_ · H_2_O)^17–21^, exfoliation and stabilization occurs through the intercalation of polyoxyalkyleneamine with a molecular weight of 1 kDa (Jeffamine M1000). In case of equivalent amounts the obtained solid comprises an *α*-ZrP weight fraction of just 13 percent. Despite low to moderate *α*-ZrP loadings the coatings’ performance is expected to be impaired by the hydrophilic and soft brush polymer.

In order to circumvent those drawbacks as well as the use of organic solvents, the exfoliation into waterborne, liquid crystalline phases appears to be an attractive route. Nematic, columnar and smectic phases of electrostatically stabilized, charged nano-platelets have been described for different inorganic 2D frameworks like clays^24–26^, gibbsite^27,28^, layered double hydroxides^29^, titanate^30^, phosphatoantimonic acid^31,32^ as well as *α*-ZrP^33,34^. Contrary to phosphatoantimonic acid, *α*-ZrP does not swell and exfoliate into water unless its apical P-OH groups (Fig. 1c) become deprotonated through the action of a base. Thus, the discotic, *α*-ZrP based liquid crystal phases studied in the group of Cheng^33–35^ were based on tetrabutylammonium (TBA) hydroxide treated *α*-ZrP. Sun *et al*. reported nematic (*N*) and smectic (*S*) phases with *I-N* and *N-S* transitions at volume fractions *ϕ* around 0.01 and 0.06, respectively^33^. However, the authors considered two TBA layers to contribute to the platelets’ thickness *t* which reduces the particles’ aspect ratio ζ = *L*/*t*, with *L* being the lateral size, and poses questions with regard to the given volume fractions and the XRD derived repeat distances in *N*-*S* dispersions.

Here we present the exfoliation of *α*-ZrP platelets into aqueous dispersion through the intercalation of alkanol amines and report on their hierarchical order which comprises empty liquid. Additionally we demonstrate how these particle-free interstices can be filled with polymer colloids of largely varying sizes, enabling the formulation of organic coatings that dry into anisotropic, *α*-ZrP based composite films. Furthermore, we show that the colloidal state of the exfoliated platelets is rooted in the hydrolysis of the *α*-ZrP which is known to accompany its intercalation by amines^36–38^. Finally, our results add new facets to the pioneering work of Kaschak *et al*. who studied the hydrolysis of *α*-ZrP in the course of TBA intercalation^39^.

## Results

**Figure 2:**
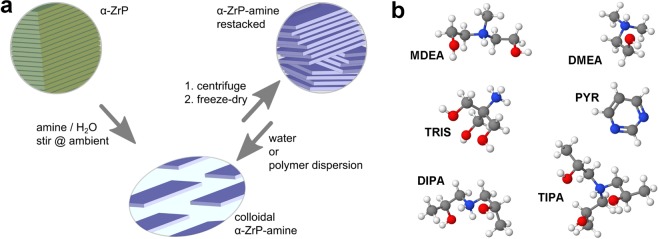
(**a**) Scheme for *α*-ZrP exfoliation and colloidal processing. The centrifugation yields a lower *α*-ZrP-MDEA enriched phase which has a yield point and is thus denoted “gel-phase” in the text. If not noted otherwise, fresh samples from redispersed, freeze-dried *α*-ZrP-MDEA are scrutinized. (**b**) 3D structures of *α*-ZrP ammonium counter ions and pyrimidine (cf. Table 1, blue N, red O, grey C, white H).

The general experimental process is summarized in Fig. 2. Pristine *α*-ZrP is obtained via the reflux method in 6M H_3_PO_4_ with decimolar zirconyl chloride and exfoliated with equivalent amounts of amine into a homogeneous pristine dispersion (*tel quel*). Centrifugation and freeze-drying of the gelatinous sediment provides a stock of ammonium intercalated *α*-ZrP in an overall yield of 40% with respect to the zirconium source. Discarding the supernatant enriches larger platelets and reduces the amount of hydrolysis byproducts (cf. chapter *α*-ZrP hydrolysis). However, after more than three successional cycles of this fractionation the dried matter becomes indispersible in water whereas it readily disperses in water after the first cycle without the need to apply shear or another energy input like ultrasound. This points to a loss of ammonium counter ions in the course of the fractionation and/or the freeze-drying step. Amine uptake and ammonium retention after a single centrifugation, freeze-drying cycle is determined by thermal and elementary analyses with consistent results (Table SI-1). Variations for the different amines (Table 1) are discussed in the next chapter. Dynamic light scattering and TEM analysis (vide infra) consistently reveal a mean particle size of 300 nm which is in accord with the particles seen in SEM pictures of pristine *α*-ZrP (Fig. S1).

**Table 1:** Amines used for *α*-ZrP exfoliation, listed according to increasing basicity (pK_*a*_ of the conjugated acid) together with their boiling temperature and water miscibility (cf. SI).

Amine	code	pK_*a*_	b.p. [°C]	solubility in H2O [g/100 g]	retention (%)	*d* [nm]
pyrimidine (equiv./excess)	PYR	1.1	123	misc.	11/55	0.76/1.13
triethanol amine	TEA	7.7	335	misc.	n.d.	1.48
triisopropanol amine	TIPA	7.9	306	500	78	1.65
tris(hydroxymethyl)aminomethane	TRIS	8.1	288 (dec.)	68	91	1.76
methyldiethanol amine	MDEA	8.6	247	misc.	75	1.44
diisopropanol amine	DIPA	8.9	249	misc.	82	1.68
dimethylethanol amine	DMEA	9.2	162	misc.	n.d.	n.d.

Amine retention is calculated from elementary analyses for carbon in the freeze-dried gel-phase in relation to the theoretical maximum (*α*-ZrP · 2 “amine”). The repeat distance *d* of stacked *α*-ZrP layers is obtained from powder XRD analysis (Fig. SI4).

### Amine intercalation and restacking

Amines were chosen according to their basicity in order to suppress hydrolysis, their molecular weight in order to impart lowest possible weight fraction of the counter ion into a formulation and their functionality in order to avoid nucleophilic attack on Zr or polymers of a formulation (Table 1 and Fig. 2b). Gao and coworkers reported that mono hydroxy alkyl amines (C2–C4), despite being less basic, hydrolyse *α*-ZrP more severely than their corresponding alkyl amines^38^. They interpret their results with a higher degree of intercalation of the more polar alkanol amines. Thus, tertiary amines with two or three hydroxylated alkyl groups like TEA, TIPA and MDEA appear promising candidates, whereas TRIS and DIPA as primary and secondary amines respectively may impart nucleophilic attack on zirconium^40,41^. PYR as least and DMEA as most basic amines were included in this series as reference points with regard to *α*-ZrP hydrolysis (cf. chapter hydrolysis). Equivalent amounts of PYR, that is considered as a mono-basic compound which is known to be reluctant to intercalate^42^, basically leave *α*-ZrP stacking intact (*d* = 0.76 nm). However, a tenfold excess pushes its uptake and the found repeat distance is in good agreement with that of a reported sol-gel derived hybrid material with less intercalated PYR^43^. DMEA is a commonly used amine for the neutralization of carboxylic acid groups of polymers in order to stabilize their colloidal particles in water against flocculation (cf. filling of empty liquid).

Taking into account rather similar volatilities but varying water miscibilities of the listed amines in the row TIPA to DIPA (Table 1), the different amounts of retained amine are apparently reflecting different dissociation equilibria between surface near ammonium counter ions of the *α*-ZrP platelets and the corresponding amine in the surrounding liquid. However, we can not rule out different degrees of sublimation during freeze-drying and specific interactions between the ammonium counter ions or between them and the platelets or the hydrolysis byproducts.

Indeed, albeit being different, the amines’ retentions as well as the repeat distances within the freeze-dried solids (TIPA to DIPA) are strikingly similar in comparison to largely varying values that were observed within the homologous series of methyl(ethyl)-, dimethyl(ethyl)- and trimethyl(ethyl)amines^44,45^. Within the ethyl series the accommodated amount and the interlayer spacing decrease with an increasing number of alkyl groups: 100, 50, 25% and 1.47, 1.26, 1.26 nm, respectively. Similar trends, although less consistent for the gallery heights, were found within the methyl series^44^. It is beyond the scope of this article to determine the molecular interstitial arrangements of the ammonium cations investigated here. Nevertheless, we assume a bilayer assembly, probably involving hydrogen bonding with co-intercalated water. In case of TIPA and in particular DIPA additional reflexes in SAXS of their colloidal states (Fig. SI5) as well as peaks in their corresponding powder XRD (Fig. SI4) may indicate more complex intra-gallery chemistry involving zirconium species from the hydrolysis (cf. chapter hydrolysis).

In the following, our investigations focus on MDEA exfoliated *α*-ZrP although TEA was found to produce similar colloidal states as MDEA. However, TEA derived dispersions severely discolour over weeks to months under ambient conditions which was not investigated here. Nevertheless, tetra(triethanolamine)zirconium (CAS 101033-44-7) is claimed as an adhesion promoter, commercialized as ZCA-TEAZ for catalytic (trans)esterifications, used as a cross-link agent for carboxymethylcellulose based hydraulic fluid^46^ and reported to circumvent oxygen inhibition in radical acrylate photopolymerization^47^. Furthermore, zirconium amide complexes are known to react with oxygen to form peroxo complexes^41^. This leaves unanswered questions to the authors why swapping one hydroxyethyl group for a methyl group entirely suppresses the formation of chromophores.

### *α*-ZrP hydrolysis

Hydrolysis of the platelets during their exfoliation process with MDEA was monitored over time by taking samples which were centrifuged and their supernatants analyzed for their Zr and P content via ICP-OES with a sample from the pristine batch as a control. Within the first 48 hours 11.5 ± 0.9 mol-% P and 6.6 ± 0.6 mol-% Zr were constantly retrieved (Fig. SI6). The high P/Zr ratio of 3.5 ± 0.2 versus two for the control sample is plausible in the light of excess phosphoric acid in the *α*-ZrP synthesis which yields phosphate terminated rims. The result confirms data reported by Xu *et al*.^37^ with other amines. The absolute proportion of retrieved matter appears reasonable in the light of two weight-% hydrolyzed material being estimated for uniform, circular platelets (*r* = 150 nm) and a hydrolysis depth of 0.75 nm which is the mean value of the unit cell dimensions in the a-b plane (elimination of only the outermost groups).

However, after two days the concentration of P and Zr in the supernatants steeply increases up to 30 and 20 mol-%, respectively (samples taken on day 3, 15 and 30). Such an inhibition period with decreasing P/Zr ratios and increasingly scattering values is unreasonable since hydrolysis proceeds from the edges where the layered framework is coordinatively unsaturated. The results can be best understood by the release of smaller platelets from the colloidal assemblies (cf. next paragraph) which stay in the supernatants. We note that the intercalation should be completed within one hour according to XRD analysis on quenched samples (not shown), although, strictly speaking it merely proves the absence of sufficiently thick stacks to cause diffraction peaks of pristine *α*-ZrP.

**Figure 3:**
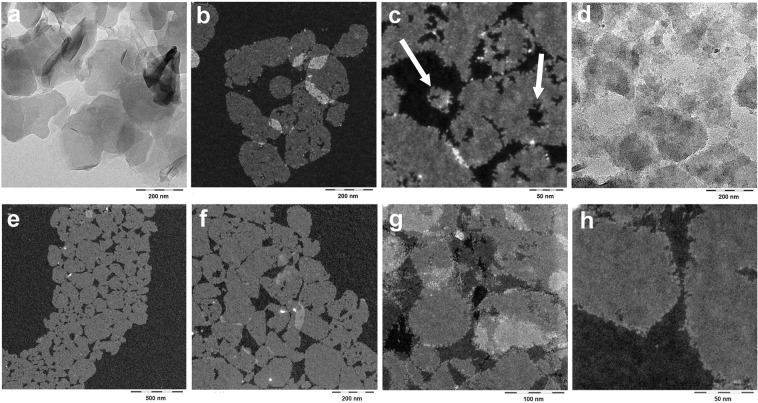
TEM images of dried diluted dispersions. (**a**–**c**) MDEA exfoliated *α*-ZrP: bright field (**a**) versus dark field (**b**,**c**). (**d**) Bright field image of DMEA exfoliated *α*-ZrP. (**e**–**h**) Dark field images of MDEA exfoliated “*273 tel quel*” after TFF. Scale bars are 500 nm (**e**), 200 nm (**a**,**b**,**d**,**f**), 100 nm (**g**) and 50 nm (**c**,**h**).

Hydrolysis is also visualized by TEM images. The different extents of particle erosion as a function of the amines’ basicity are obvious in bright field images of *α*-ZrP-MDEA versus *α*-ZrP-DMEA (Fig. 3a vs. d). By adopting a quantitative sample preparation of the diluted exfoliation products and applying high-angle annular dark field (HAADF) imaging, the added diffraction contrast overcompensates a loss in mass thickness contrast and uncovers details of the morphology of predominantly single to few layer thin stacks. This is shown for *α*-ZrP-MDEA (Fig. 3a vs. b,c). Platelets appear pixelated and are apparently composed of 2–5 nm large grains that are resolved at the particles edges (cf. Fig. 3c). However, these are distinctly smaller than the crystal domain size *L* = 25–30 nm which can be estimated from the full width half maximum (FWHM) XRD peak analysis for the in plane reflexes *110* and *020* according to Scherrer^48,49^ (Fig. SI2). Intriguingly, the platelets exhibit holes and fjords of that dimension, while similarly sized flakes are found around them (arrows, Fig. 3c). Presumably complementary flakes and lacunae in conjunction with a consistent crystal domain size will be discussed with respect to *α*-ZrP synthesis (vide infra). We stipulate that the formation of such tiny flakes and their slow release from the platelets’ assembly are the cause for the apparent increase of hydrolysis after the induction period.

Finally, with TRIS, TIPA and DIPA exfoliation, similarly eroded platelets are displayed by TEM analysis, with the highest degree of degradation for the DIPA derived sample (Fig. SI3). In conjunction with additional peaks in SAXS of the dispersion (Fig. SI5) and powder XRD of the restacked solid (Fig. SI4), these results indicate that amine intercalation may involve reactions which can not solely be explained with hydrolysis in a basic milieu. However, these chemical pathways are not scrutinized here. Finally, PYR intercalation leaves the inorganic framework basically intact (Fig. SI3j–l).

### Hydrolysis suppression & purification

Hydrolysis can be mitigated through gradual amine addition in order to avoid temporaryly and locally high base concentration as well as by cooling, which should decelerate chemical attack according to Arrhenius’ law. The temperature dependence for the intercalation process is less straightforwardly a function of the diffusion in confined space^50^ and the widening of the latter^51^ which in turn depends on the host-layer flexibility^52–56^ as well as molecule-ion^57^, molecule-host^58^ and ion-ion^59,60^ interactions. Nevertheless, both parameter changes were adopted in one batch with dropwise MDEA addition (27 g/h) to a cooled *α*-ZrP dispersion (0 °C, denoted “*273*”). Agitation during the addition needs to be increased in order to avoid temporary formation of an amine-rich layer on the aqueous phase. Despite cooling, the mixture heats up to 5.7 °C during the exfoliation. Analysis for soluble hydrolysis products in the supernatants of specimens taken over the first four hours reveals that hydrolysis is suppressed by a factor of nearly two thirds in comparison with the standard procedure while the P:Zr ratio stays unchanged above three (not shown).

In order to remove the hydrolysis products from the platelets, tangential flow fractionation (TFF) was applied on the diluted pristine dispersion (*tel-quel*) as well as on the diluted gel-phase (*gel*) from both processes (*273*, *standard*). The concentrations of phosphorus and zirconium in the permeates were monitored by ICP-OES analysis. All four samples yielded similar results and the retrieved amounts of P and Zr are representatively given for “*273*, *gel*” in Table 2. Distinctly high P:Zr ratios point to chemisorption of zirconium species on the cellulose membrane which is in accordance with earlier observations on thin film chromatography of metal ions^61–63^. However, swapping the membrane for polyethersulfone still discriminates Zr and even with sixfold wider pores the P:Zr-ratio still exceeds the *α*-ZrP-stoichiometry by a factor of five. Pore sizes are calculated to be 2.2 nm and 13.2 nm according to *R* = 0.066*M*^13^ for the radius of a protein sphere with *ρ* = 0.73 *cm*^3^/*g* and *M* = 5 kDa and 1,000 kDa, respectively^64^.

**Table 2:** Phosphorus and zirconium from *α*-ZrP-MDEA samples “*273*, *gel*”, retrieved in the permeates after TFF over cellulose (Ultracel^®^) and polyethersulfone (Biomax^®^) membranes respectively (cf. Table SI-2).

Membrane	Pore Size	P [%]	P/Zr
Ultracel^®^	5 kDa	4.6 (8.7)	160* (1.6)
Biomax^®^	5 kDa	2.7 (4.3)	31 (2)
Biomax^®^	1,000 kDa	2.4 (4.1)	10 (2)

Parenthesized numbers denote the P contents and P/Zr ratios from the washing solutions of a subsequent flush with deionized water. The asterisk denotes that the given P:Zr ratio declines over the fractions from 160 to 70.

Phosphate thus permeates the membranes to a significant proportion with MDEA-H^+^ counter ions, since the predominant species is ${{\rm{H}}{\rm{P}}{\rm{O}}}_{4}^{2-}$ in the basic permeates with pH ~9. Despite having compensated a pH drift in the retentate through the addition of 1 weight-% MDEA, further 12% of the initial MDEA equivalents are missing in the freeze-dried products according to elementary analysis.

The pore size dependent retention of zirconium apparently indicates the presence of zirconium comprising species that are larger than the exclusion size of 2.2 nm. Without claiming chemical congruency, polyoxometalates like Keggin-type anions are known to adsorb on soft, non-ionic surfaces^65,66^. Analogous Zr-species may thus play a role in membrane fouling through interactions with both the membrane and the *α*-ZrP platelets (cf. values after flush, Table 2). Furthermore, the separation of phosphate through TFF tends to promote platelet aggregation which is detected by the volume based evaluation of dynamic light scattering (DLS, Table 3). This aggregation is also reflected by increased proportions of laterally extended layers on the sample holder for TEM analysis (Fig. 3e,f) and in small angle X-ray scattering (SAXS) (cf. Fig. 6a).

**Table 3:** *α*-ZrP-MDEA particle sizes, measured with dynamic light scattering (DLS), as a function of the exfoliation conditions (“*273*” & standard), the colloidal processing of both reaction mixtures (“*tel*-*quel*” & “*gel*” fraction) and the impact of the TFF purification.

Sample		Z (*d*)	PDI	V (*d*)	D90 (*d*)
*gel* (*N* = 4)	$\bar{x}$	347	0.23	449	765
	*s*	12	0.03	19	89
*tel quel* (*N* = 4)	$\bar{x}$	339	0.24	712	1,432
	*s*	16	0.03	730	1,812
*after* TFF (*N* = 8)	$\bar{x}$	377	0.39	1,192	2,597
	*s*	51	0.07	916	1,993

All samples were freeze-dried, dispersed in water to 0.1 weight-% and the dispersions were aged for two weeks before two measurements took place. Arithmetic means ($\bar{x}$) and standard deviations (*s*) of the particle diameters (*d* [nm]), poly-dispersity indices (PDI) are given for each set of samples with *N* denoting the number of measurements.

### Colloidal state

Aqueous dispersions from the freeze-dried gel-fraction of *α*-ZrP-MDEA that was obtained under ambient conditions have been scrutinized for the colloidal state of the platelets as a function of their concentration. In the presented range from 2.5 to 20 weight-% those dispersions with higher loadings stay homogeneous over at least 15 months, whereas slow phase separation takes place over several months with 5 weight-% and less (cf. chapter “filling of the empty liquid”).

**Figure 4:**
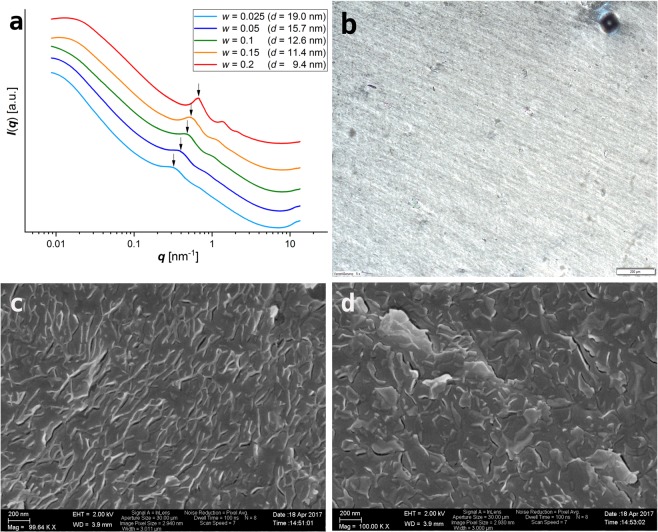
Dispersion of MDEA exfoliated *α*-ZrP. (**a**) Vertically shifted SAXS curves for increasing weight fractions (*w*) of *α*-ZrP-MDEA. Arrows point to the maximum of the coressponding interlayer distance (*d*) in each dispersion. (**b**) Birefringence in an OPM image of the dispersion with *w* = 0.2 (scale bar 100 *μ*m). (**c**,**d**) Cryo-SEM images of the dispersion with *w* = 0.2 (scale bars 200 nm) show edges of preferentially aligned tactoids that consist of few layers of stacked *α*-ZrP platelets which are partially resolved in tactoids protruding the fracture surface (**d**).

The interlayer distance decreases with increasing weight fractions from 19 nm (*w* = 0.025) to 9.4 nm (*w* = 0.2) according to observed intensity maxima in small angle X-ray scattering (Fig. 4a). The latter become more pronounced with increasing concentration and lamellar alignment is unambiguously displayed for weight fractions above *w* = 0.1 by three harmonic humps with *q*-values in the ratio 1:2:3. The broadness and low number of harmonics point to a low number of coherently scattering platelets. This indicates a small number of stacked layers possibly in conjunction with a poor stacking order that could be caused by fluctuating spacings or by undulated layers like in lipid bilayer membranes^67–69^. Although bending appears less likely for rigid, inorganic frameworks it is worth noting that Xia *et al*. presented crumpled exfoliated *α*-ZrP sheets in TEM images^70^. Nevertheless, the scattering curve (*w* = 0.2, Fig. 4a) can be approximated for few layers in the scattering domain using either the paracrystalline or the modified Caillé approach (Fig. SI7).

The stacked platelets are aligned in nematic order according to a birefringence texture in cross polarized light (Fig. 4b) which appears as a hybrid of those that are attributed to nematic^24^ and smectic^6^ phases. This is corroborated by cryo-SEM images which show preferentially aligned edges of the dispersed matter (Fig. 4c,d).

The volume fraction of the neat *α*-ZrP layers (*ϕ*_*ZrP*_) can be obtained from the weight fraction (*w*) of the *α*-ZrP-MDEA by taking into account the experimentally determined proportions of MDEA and adsorbed water in the freeze-dried stock material and the density of the inorganic framework (*ρ*_*ZrP*_ = 2.76). For *w* = 0.2 a volume fraction of *ϕ*_*ZrP*_ = 0.044 is calculated which is distinctly lower than the one of a hypothetical layered material of thickness *t* which is stacked into a lamellar phase with a lateral area coverage of *ϕ*_2*D*_ ≈ 1 and a repeat distance *d*. Applied to the 20 weight-% *α*-ZrP-MDEA dispersion (*t*_*ZrP*_ = 0.68 nm, *d* = 9.4 nm) such lamellae would occupy a volume fraction of *ϕ*_*lam*_ = *t*/*d* = 0.072. However, tactoids that are built from stacked *α*-ZrP layers are more realistic than an area coverage of one. Even for the packing of small cylinders (tactoids) in a big cylinder (container) the theoretical maximum area coverage of an infinite hexagonal array would be $\rho =\pi /2\sqrt{3}=0.907$ (http://oeis.org/A093766)^71^. The volume fraction of the tactoids can be calculated from *ϕ*_*ZrP*_ according to *ϕ*_*tac*_ = *ϕ*_*ZrP*_ (1 + (*d* − *t*)/*t*). For the *α*-ZrP-MDEA dispersions with *w* = 0.1 and *w* = 0.2 tactoid volume fractions of *ϕ*_*tac*_ = 0.39 and *ϕ*_*tac*_ = 0.61 are obtained, respectively.

**Figure 5:**
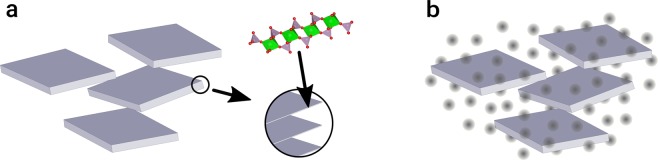
(**a**) Model of hierarchical order of nematically aligned few layer thin *α*-ZrP-MDEA tactoids. (**b**) Polymer colloids are populating the otherwise empty liquid between the tactoids.

We emphasize that the real colloidal picture is likely more complex, not least due to size dispersity and irregular particle shape. However, the present results can be understood as a hierarchical assembly of *α*-ZrP platelets that stack into few layer thin tactoids which in turn arrange into nematic order (Fig. 5). The structuring tactoids are not space filling and leave empty liquid for the accommodation of other colloidal particles (cf. chapter “filling of the empty liquid”).

### Impact of electrolytes

The dispersed, anionic *α*-ZrP platelets are stabilized by electrostatic repulsion between equally charged surfaces. However, in favour of the hierarchical assembly they do not evenly occupy the sample volume which suggests the action of an attractive component. It is well known that electrolytes screen surface charges of dispersed particles which decreases the repulsion force.

**Figure 6:**
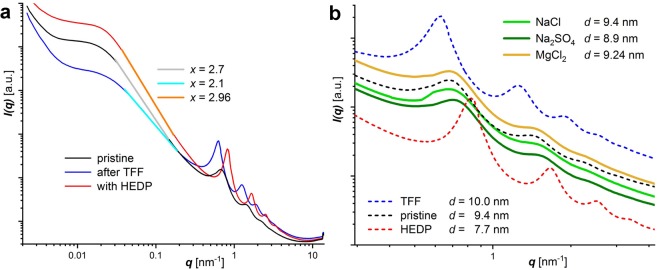
SAXS curves of *α*-ZrP-MDEA dispersions (*w* = 0.2). (**a**) Pristine dispersion versus the effect of added sodium HEDP (0.019 M) and TFF purification, respectively. Linear fits are superimposed and the slopes for *I*(*q*) ∝ *q*^−*x*^ are included. (**b**) Zoomed SAXS section for the harmonic peaks of the *α*-ZrP tactoids with the curves shown in (**a**) as dashed lines in comparison to those obtained from the pristine dispersion with added NaCl (same [Na] as with HEDP), Na_2_SO_4_ ([SO_4_] = [HEDP]) and MgCl_2_ ([Mg] = [HEDP]). Vertically shifted curves in (**b**).

It is thus expected that the inter-layer distance for a given volume fraction will vary in the presence of dissolved salts like NaCl, Na_2_SO_4_ and MgCl_2_. However, as shown by SAXS records (Fig. 6b) of *α*-ZrP-MDEA dispersions with *ϕ*_*ZrP*_ = 0.044 the platelets stacking found with the original salt-free dispersion is basically unchanged by the cations and chloride, whereas the oxoanion, which is known for its affinity for zirconium and the lability of the reaction products in aqueous phase^72–74^, causes a moderate decrease of the repeat distance. Conversily, TFF purification over Ultracell (5 kDa) and the addition of sodium bisphosphonate (1-hydroxyethylidene-1,1-diphosphonate, HEDP) distinctly shift the peak positions to lower and higher *q*-values, respectively. The widening of the interstitial phase after ultrafiltration can be attributed to the removal of hydrolysis products, leading to a net reduction of intrinsic electrolytes. HEDP as a chelating ligand is known to form a variety of monomeric to oligomeric complexes with zirconium^75–77^ and presumably reacts with preexisting zirconium hydrolysis products in the interstitial space and possibly modifies or erodes the platelets’ rims via complex formation with exposed zirconium sites. Besides charge screening, the interaction of large, super-chaotropic reaction products with the ammonium counter ions may as well alter the platelets’ effective charge density (cf. discussion).

Indeed, the scattering in the low *q*-range supports the findings. The intensity in the linear sections 0.03(4) < *q* < 0.2 decays as *I*(*q*) ∝ *q*^−*x*^ with *x* = 2.1, 2.7, 2.96 for the purified, the pristine and the HEDP doped dispersion, respectively (*R*^2^ ≥ 0.9997). A slope with *x* = 2 is expected for 2D objects with a smooth interface, whereas larger *x*-values are attributed to mass- and surface-fractal objects. Although the use of fractal models appears mathematically questionable in the light of limited scaling orders in the colloidal domain^78^ it is widely accepted. In accordance with our TEM findings (Fig. 3c), the discrimination of zirconium in ultrafiltration (Table 2) and the shifted repeat distances in SAXS (Fig. 6b), increasing *x*-values larger than two can be interpreted in a physically meaningful way as rugged interphases that are formed from nano-metric hydrolysis products (Zr-based cluster). The transitions to the plateaus around *q* ≈ 0.02 nm^−1^ correspond to the platelets’ size of ≈300 nm. With further decreasing *q*-values the plateaus end and an increasing scattering intensity indicates the presence of larger objects which can be associated with laterally extended tactoids. The inflection point at *q* ≈ 0.005 nm^−1^ corresponds to micrometer sized objects in real space which is in agreement with the observations in dynamic light scattering (DLS, Table 3) and TEM analysis (Fig. 3e,f).

### Filling of the empty liquid

The empty liquid between hierarchically ordered *α*-ZrP platelets should be able to accommodate polymer colloids of varying particle size. In practice the freeze-dried gel phase (Fig. 2a) is dissolved in aqueous polymer dispersion instead of water. To demonstrate the broad applicability of this approach, polymer colloids have been chosen that differ with regard to polymer chemistry, molecular weight and functionality. They are listed according to their particle size in water in Table 4. All are anionically stabilized through DMEA neutralized carboxylic acid groups and with the exception of PUR-*μ*-gel they bear hydroxyl groups intended for chemical cross link formation. Solid contents vary according to synthesis specific process parameters and aspects like coagulation. We note that PES and PUR_*a*_ are identical to PES_h_(PES_NB_) and PUR_*h*_ that have been used in previous work^8,29^. They are soft (T_*g*_ ≤ 273 K) and amphiphilic due to fatty acid based monomers and they are the only dispersions which provide 2-butoxyethanol (BE) as a co-solvent with 6 and 20 weight-% in the delivery state of PUR and PES, respectively. PUR_*b*_ is less hydrophobic than PUR_*a*_ due to less dimer fatty acid moieties in the backbone. PUR-PAC is a commercial hybrid product (cf. methods). The soft PUR-*μ*-gel (T_*g*_ ≤ 273 K) is cross linked through *in-situ* addition reaction on isocyanate terminated chains and comprises roughly 15 weight-% of extractable matter (THF, 24 h, r.t.). PAC-*μ*-gel is a core-shell latex polymer of (meth)acrylic monomers with a cross-linked core and a glass transition of (T_*g*_ ≤ 273 K).

**Table 4:** Used aqueous polymer dispersions.

Polymer	M_*n*_	M_*w*_	${\bf{RC}}{{\bf{O}}}_{{\bf{2}}}^{{\boldsymbol{-}}}$	ROH	NYC	*d*
	[kDa]	[kDa]	[meq/g_*NVC*_]		[%]	[nm]
PUR_*b*_	5	14	0.66	0.87	40	20
PUR_*a*_	14	51	0.45	0.23	27	27
PES	2	21	0.58	1.25	60	65
PUR-PAC	1	10	0.25	n.d.	36	85
PUR-*μ*-gel	micro-gel		0.32	0	40	188
PAC-*μ*-gel	micro-gel		0.31	0.3	27	240

Molecular weights refer to PMMA standards in size exclusion chromatography (SEC), carboxylate and hydroxyl contents refer to the non-volatile matter (NVC, after 1 h at 130 °C). Particle sizes were measured in diluted dispersion (0.1 w-%) by DLS.

### Macroscopic phase behaviour

**Figure 7:**
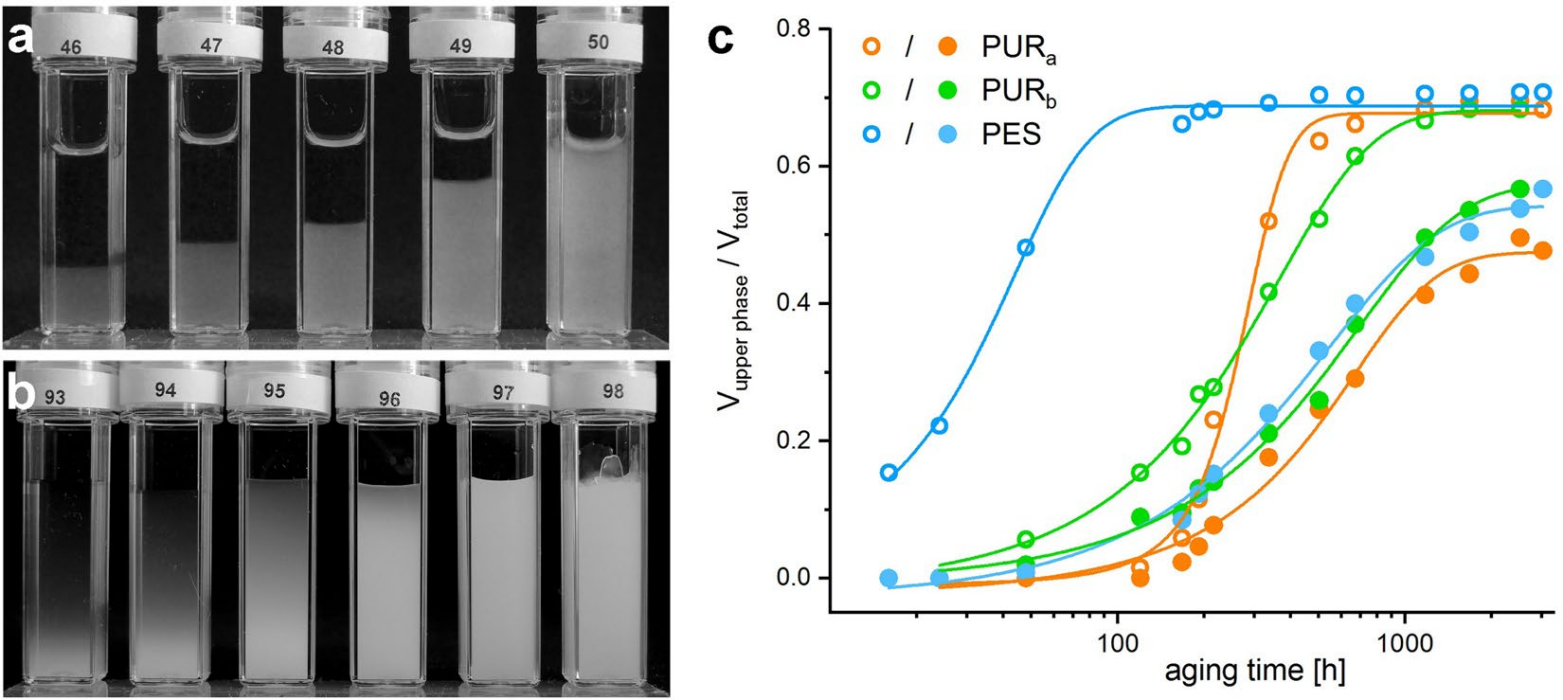
Cuvettes with increasing *α*-ZrP-MDEA weight fractions (*w*), from left to right 0.005, 0.01, 0.025, 0.05 and 0.1 as well as 0.2 in cuvette No. 98. Photographed after 69 weeks (**a**) in admixture with PUR_*b*_ (*w* = 0.2) and after 61 weeks (**b**) without polymer. (**c**) Phase separation kinetics for the PES, PUR_*a*_ and PUR_*b*_ bearing dispersions (*w*_*poly*_ = 0.2) with two different *α*-ZrP-MDEA weight fractions: *w* = 0.005 (open symbols) and *w* = 0.01 (solid circles).

The phase behaviour of dispersions with a constant polymer weight fraction (*w* = 0.2) and increasing *α*-ZrP-MDEA weight fractions (*w* = 0.005, 0.01, 0.025, 0.05, 0.1) was monitored over time. With polymer particle sizes larger than 70 nm any changes are barely visible regardless of the type of illumination due to light scattering. Less scattering polymer colloids allow for the monitoring of the phase separation into an upper isotropic and a lower ordered phase as shown exemplaryly for PUR_*b*_ (Fig. 7a). The phase separation is accomplished within several weeks and the absence of any further visible change after 69 weeks suggests that phase equilibria have been reached. Albeit, the kinetics differ depending on the polymer colloid and the volume fraction of the platelets (Fig. 7c). For the highest concentration (*w* = 0.1) the sample volume is almost completely occupied by the ordered phase. However, in the absence of any polymer the phase separation is much slower and even after 61 weeks phase equlibria are not achieved according to diffuse gradients in opaqueness instead of sharp phase boundaries (Fig. 7b). No change can be seen with the highest volume fractions (*w* = 0.1) and (*w* = 0.2, cuvette 98). The latter has been added in order to demonstrate a shift to non-fluid behaviour at higher concentrations.

**Figure 8:**
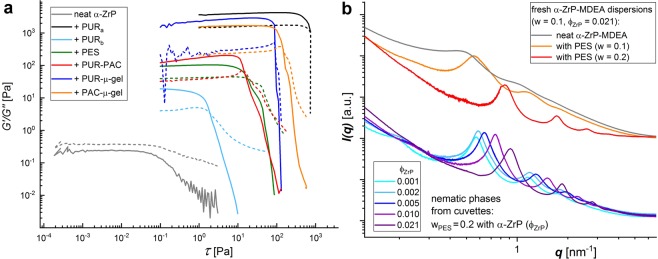
(**a**) Amplitude sweeps of *α*-ZrP-MDEA dispersions (*w* = 0.1, *ϕ*_*ZrP*_ = 0.0212), neat and in combination with polymer colloids (*w* = 0.2). Solid and dashed lines depict the storage (G′) and the loss modulus (G″), respectively. (**b**) Zoomed sections of SAXS curves from fresh *α*-ZrP-MDEA dispersions (*w* = 0.1), neat and in admixture with PES (*w* = 0.1 & 0.2), in comparison with curves from the aged, settled nematic phases from sibling cuvettes of those shown in Fig. 7a (former curves are shifted vertically).

Nevertheless, the rheology of the viscous liquid of the *α*-ZrP-MDEA dispersion (*w* = 0.1) which is dominated by the loss modulus (G″) changes into elastic fluids if polymer colloids are populating the otherwise empty liquid. This is evidenced by amplitude sweeps which reveal a dominant storage modulus (G′) and a yield point (Fig. 8a). The varying strength of the gel-like dispersions under deformation until G′ and G″ intersect and their different viscosity which scales with the moduli obviously do not correlate with the particle size or another singular feature of the polymer colloids under scrutiny. Still, with regard to their comparable dimensions, particle jamming appears to be a plausible explanation for the similarities between the two micro-gels, as well as between PES and PUR-PAC, respectively. In contrast, the difference between PUR_*a*_, PUR_*b*_ and PES should be due to peculiar effects (vide infra).

### Colloidal states

SAXS records from the aged lower phases of the cuvettes and of fresh, homogeneous dispersions suggest that the polymers do not intercalate (Figs 8b and SI8). On the contrary, the peaks caused by the platelets stacking become sharper and shift to larger *q*-values. In the fresh samples of *α*-ZrP-MDEA (*w* = 0.1) with PES the repeat distances *d* decrease from 12.6 nm (no PES) over 11.2 nm (*w*_*PES*_ = 0.1) to 7.5 nm (*w*_*PES*_ = 0.2) and in the lower phases from the cuvettes with *w*_*PES*_ = 0.2 the *d*-values are distinctly smaller than those of the polymer free dispersions (cf. Fig. 4) with 10.9 vs. 19 nm and 6.4 vs. 9.4 nm for the highest and lowest *α*-ZrP-MDEA weight fractions, respectively. This can be explained by depletion forces, in other words osmotic pressure, which scales with the polymer loading at a given *α*-ZrP concentration. We note that the ordered phase in the sample with the highest *α*-ZrP-MDEA content (*w* = 0.1) basically did not shrink during aging but still displays a 0.6 nm smaller layer spacing compared to the fresh dispersion, which points to slow consolidation processes in the colloidal system. Furthermore, broad humps at *q* = 0.2 nm^−1^ in the cuvette samples with the two lowest *α*-ZrP concentrations can be attributed to polymer colloids. Their size (*D* ≈ 31 nm) is roughly half of the hydrodynamic volume measured with DLS in the neat, highly diluted PES dispersion (*D* = 65 nm). Possible explanations comprise distribution equilibria of PES, a diffuse shell and core morphology of PES particles and counter ion scrambling (MDEA/DMEA). Nevertheless, no major changes in the scattering curves of *α*-ZrP platelets have been observed in the presence of PES and PUR_*a*_ with exclusively MDEA or DMEA based counter ions, respectively (not shown).

**Figure 9:**
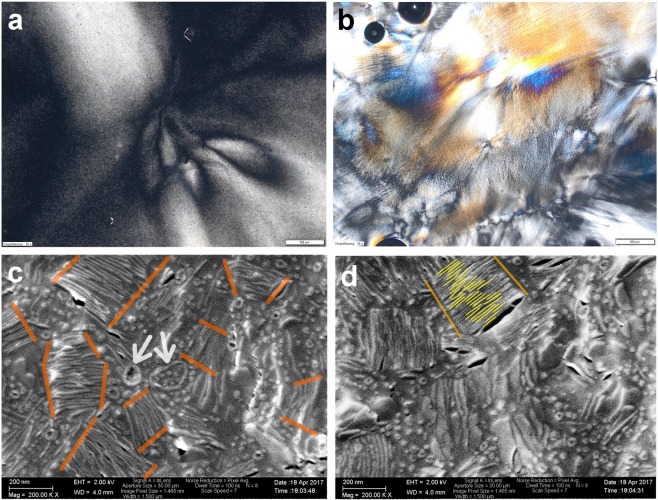
OPM pictures from *α*-ZrP-MDEA dispersions (*ϕ*_*ZrP*_ = 0.0212) comprising PES (**a**) *w* = 0.1 and (**b**) *w* = 0.2 (scale bars 100 *μ*m). (**c**,**d**) Cryo-SEM images from *α*-ZrP-MDEA (*ϕ*_*ZrP*_ = 0.0212) with PES (*w* = 0.06) (scale bars 200 nm). (**c**) Orange lines highlight columnar like piling of *α*-ZrP layers and arrows point to PES vesicles. (**d**) Yellow lines highlight arbitrarily chosen edge sections of individual *α*-ZrP layers that are piled into a column.

The increased distinctness of scattering peaks may reflect a better platelet alignment due to narrower interstitials or an enhanced correlation length caused by aggregated tactoids. Indeed, a changed colloidal platelet arrangement in the presence of PES is indicated by another birefringent texture in cross-polarized microscopy (Fig. 9a,b), as well as cryo-SEM pictures which reveal platelets that are piled into stacks with 20 to 30 layers (Fig. 9c,d). A spacing of 10 ≤ *d* ≤ 15 nm can be estimated, which is in reasonable agreement with the repeat distance from SAXS (Fig. 8). Furthermore, in accordance with previous work on the self-assembly of PES (=*PES*_*NB*_)^79^ in aqueous phase, vesicles (arrows, Fig. 9c) are displayed in the images. Such a transition from nematically aligned tactoids to their stacking into columns may not be linked to specific features of PES, but could be a general phenomenon caused by the presence of anionically stabilized polymer colloids as suggested by similar SAXS results obtained with the other polymers listed in Table 4 (Fig. SI8).

In fact, González Garcia *et al*. have recently reported on the multi-phase coexistence in discotic colloid dispersions that is driven by the osmotic pressure caused by non-adsorbing polymers^80^. They demonstrated that the isotropic (I) to nematic (N) to columnar (C) phase transitions and the regions of I-N-C coexistence in the system’s phase diagram are depending on the aspect ratio of the 2D particles (∧ = *t*/*L*), the polymer to platelet size ratio (*q* = *d*_*dep*_/*L*, with *d*_*dep*_ the depletant diameter), the 2D particle volume fraction (*η*) and the volume fraction of the polymer colloids (${\varphi }_{d}^{S}$). Since the polymer colloids in our study are stabilized through carboxylate groups, it is reasonable to assume that they act as depletants in the sense that they do not adsorb on the equally charged platelets’ surface and do not penetrate the interlayer space. If we take our few-layer-tactoid model (Fig. 5a) as the actual 2D particles we obtain coordinates which may fall into a coexistence region that comprises a columnar phase. Assuming stacks of four *α*-ZrP-layers with *d* = 12.6 nm and *L* = 265 nm the dispersion in cuvette 50 (*w* = 0.1 *α*-ZrP-MDEA, *w* = 0.2 polymer) would be described by ∧ = (3*d* + *t*)/*L* = 0.145, *η* = *ϕ*_*tac*_ = 0.39 and ${\varphi }_{d}^{S}$ ≈ 0.2 and should thus undergo separation into coexisting I-N-C phases (cf. Fig. 4 of the referenced article)^80^. We emphasize that the input data are taken from the neat *α*-ZrP-MDEA dispersion, whereas the mixtures with polymer colloids are made directly from the common freeze-dried stock material. The values for ∧ and *η* are therefore to be considered as an egg-and-hen problem with regard to the causality. Furthermore, the piling of tactoids into columns does not proceed into the formation and separation of a columnar liquid crystalline phase. This is probably impeded by the high loading and viscosity of these mixed dispersions. Indeed, such columnar entities may form obstacles to gliding planes under imposed shear, contrary to the nematic alignment of tactoids. In conjunction with particle size depending jamming of the polymer colloids in the surrounding liquid, high viscosities and yield points (Fig. 8a) would prevent phase separation. Finally, the highly diverging viscosities between the PUR_*a*_ and PUR_*b*_ comprising mixtures are attributed to chemical interaction between geminal hydroxymethyl groups at PUR_*a*_ chain ends with neighboring zirconium sites, like those at the platelets’ rims (Fig. SI10), or within zirconium based hydrolysis products.

### Film formation

**Figure 10:**
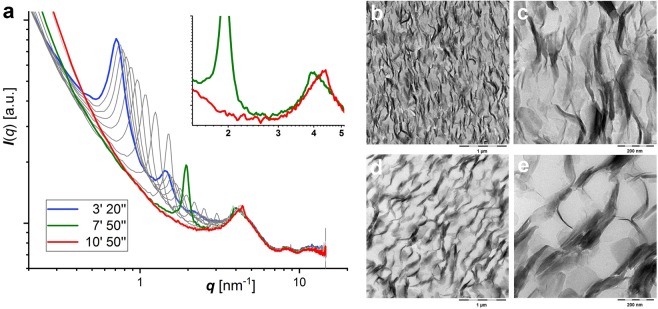
(**a**) Sections of selected SAXS curves which have been recorded in real-time of a drying film of *α*-ZrP-MDEA (*ϕ*_*ZrP*_ = 0.021) with PES (*w* = 0.1), where blue depicts the first possible measurement after sample mounting, green the last curve with a detectable harmonic peak and red the final state. A zoomed section of the latter in the insert reveals two neighbouring peaks sticking out from a broad hump of amorphous scattering caused by the polymer. (**b**–**e**) Bright field TEM pictures of cross-sections from films of the SAXS film-formation study (*w*_*ZrP*−*MDEA*_ = *w*_*polymer*_ = 0.5) with PUR-PAC (**b**,**c**) and PAC-*μ*-gel. (**d**,**e**) Scale bars are 1 *μ*m (**b**,**d**) and 200 nm (**c**,**e**).

Film formation, monitored with real-time SAXS, corroborates the colloidal states of the *α*-ZrP platelets and their reluctance to intercalate the polymers. For all investigated *α*-ZrP-MDEA-polymer combinations, the exfoliated, aligned platelets are converging during film drying as shown exemplaryly for PES in Fig. 10a. Eventually a faint peak at the position which is expected for MDEA intercalated *α*-ZrP remains at *q* = 4.36 nm^−1^ (*d* = 1.44 nm, cf. Table 1). This peak does not significantly change upon tilting of the sample (not shown), and its low intensity is due to a limited number of individual platelets and/or a poor stacking order. Indeed, a neighboring peak at *q* = 4.28 nm^−1^ (*d* = 1.47 nm) may indicate a staging phenomenon that could be caused by the scrambling of MDEA and DMEA based ammonium counter ions of the colloidal mixture. Restacking into *α*-ZrP-(MDEA, DMEA) proves the assumption that the polymers are not intercalated but act as non-adsorbing depletants instead. This is confirmed by the morphology of the films. Cryo-microtomed cross-sections are inspected with TEM (Figs 10b–e and SI9). Stacks of the inorganic material are uniformly dispersed throughout the organic matrices. Predominantly aligned in the drawing direction, the stacks’ inclinations vary and mavericks are especially frequent in the films from PES and PUR_*a*_. Despite in-plane inclinations the stacks’ thicknesses, which appear strikingly similar among the different films, can roughly be estimated to be in the order of 40 nm, which nicely matches the number of piled layers observed with cryo-SEM (Fig. 9c,d). However, the distribution and density of the collapsed columns scales with the size of the polymer colloids and thus the morphology becomes more heterogeneous with the larger polymer colloids. In the light of the different polymer types this underlines the role of their common electrostatic stabilization in aqueous phase which prevents intercalation.

## Discussion

From numerous lyotropic liquid crystal phases that are based on charge stabilized 2D nanoparticles in water and reported in the literature, *α*-ZrP exfoliated by TBA^33,34^ and phosphatoantimonic acid (PA)^32^ can be regarded as reference points for the results presented here. Despite close chemical and structural similarities between the layered materials strikingly different phase behaviour has been observed.

For *α*-ZrP-TBA platelets with decreasing aspect ratios (vide infra) isotropic-nematic^34^ and nematic-smectic^33^ phase transitions were described. We note that an inverse designation for the aspect ratio was used and that two TBA mono-layers were considered to add to the platelets thickness without specifying the calculation of their volume fraction. Taking into account that X-ray scattering is caused by the electron-density jump between the inorganic framework and the surrounding aqueous phase, their reported repeat distances should describe a different layer model. Nevertheless, based on a normalized layer thickness (*α*-ZrP-2 · TBA/*α*-ZrP = 3.97) the investigated range of volume fractions *ϕ*_*ZrP*_ reported by Sun *et al*.^33^ nearly matches the one presented here. Intriguingly, the repeat distances in the *α*-ZrP-TBA smectic phases are distinctly smaller than those of the *α*-ZrP-MDEA tactoids. Thus, *d* = 9.4 nm for *ϕ*_*ZrP*_ = 0.044 (Fig. 4a) would compare with *d* = 4.4(3.5) nm for *ϕ*_*ZrP*_ = 0.04(0.05) in Fig. 4 of the their article^33^. We agree with the assumption of Sun *et al*. that hydrophobic attraction may add to their particles’ interaction and assume that chaotropic TBA is less dissociated from the interface than the smaller, more polar MDEA-H^+^.

Conversily, phosphatoantimonate (PA = Sb_3_P_2_O_14_)^3−^) platelets with a mean particle size of 790 nm and a volume fraction of *ϕ*_*PA*_ = 0.024 adopt lamellar assembly with a repeat distance of *d* = 16.8 nm (cf. Figs 5b and 6a in ref.^32^). The spacing of 15.7 nm (*t* = 1.1 nm) exceeds the one of 11.9 nm found with *α*-ZrP-MDEA at *ϕ*_*ZrP*_ = 0.021 (cf. Fig. 4a, *t* = 0.68 nm). The authors admit that the occurence of the lamellar phase can not be fully understood on the basis of current theories. They consider interactions between patchy particles and speculate about “cation-mediated attractions between like-charged nanosheet rims”. However, no experimental evidence for the presence of such ionic species is given, whereas the preparation of the PA involved several washings and dialysis steps. It should be noted that PA has a lower charge density than *α*-ZrP and readily exfoliates into water without adding a base.

For the stacking of *α*-ZrP-MDEA into few layer thick tactoids we postulate attractive interactions that are caused by ionic, zirconium based hydrolysis products. Indeed, net attractive force between equally charged surfaces (>0.1 C/m^2^) may result from ion-ion fluctuations involving polyvalent species in the electrical double layer according to an early Monte Carlo study^81^ and recent findings with mixed Na/Ca comprising montmorillonite^82^. While such ionic species elude direct, unambiguous observation within the galleries, independent experiments of the present study unanimously suggest their existence: process parameters that leverage hydrolysis, dialysis variants which discriminate larger, Zr-based compounds and still cause an increased layer spacing, bisphosphonate and sulfate addition that leads to a shrinkage of the gallery height (contrary to ordinary electrolytes) and not least their opposing impact on the platelets’ surface smoothness and tendency to aggregation in the colloidal state, the latter two evidenced by SAXS, DLS and TEM.

**Figure 11:**
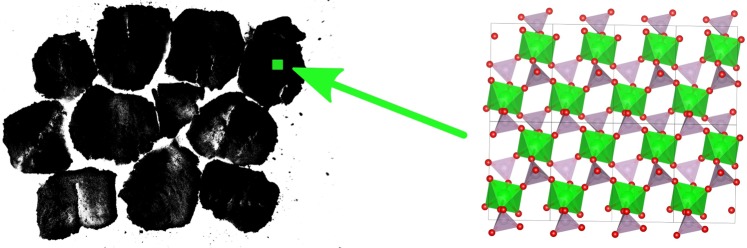
Sketched snapshot of assembled proto-*α*-ZrP flakes with sizes around 20–30 nm at the beginning of the stitching process. Eight *α*-ZrP unit cells, projected along the *c*-axis, are shown for size comparison: 2 · 9.08 (*a*) × 4 · 5.3 (*b*) = 385 Å^2^.

The apparently increasing hydrolysis over time, the concomitantly observed flakes and lacunae in darkfield TEM imaging of exfoliatied *α*-ZrP-MDEA layers in conjunction with similarly sized crystal domains in the pristine *α*-ZrP concertedly shed light on the synthesis of the latter. We stipulate lateral joining of proto-*α*-ZrP-flakes into extended *α*-ZrP platelets as sketched in Fig. 11. Stitching lines involve imperfections and/or strained bonding due to geometrical constraints which are prone to hydrolysis during exfoliation. Consequently, flakes are cut out and slowly released from the colloidal assembly of *α*-ZrP-MDEA. The stitching scenario has also been proposed by Pica *et al*. for a sol-gel *α*-ZrP synthesis on the basis of the evolution of ^31^P-NMR signals assigned to tri- and bi-dentate phosphates^83^. Further support of this mechanism is the report of Trobajo *et al*. who obtained crystalline *α*-ZrP by direct precipitation at room temperature^84^. The precipitated material had a P/Zr-ratio of 2.5–2.8 due to 15–20 weight-% H_3_PO_4_ which was not intercalated according to XRD analysis. A consistent scenario involves assembled proto-*α*-ZrP flakes which host phosphoric acid in their lateral lacunae. Under reflux conditions the acidic milieu allows for dynamic digestion and reformation processes around the rims which eventually glue flakes into layers. Our findings highlight the vulnerability of these seams towards chemical and/or mechanical stress.

In conclusion we present a novel, scalable route to ordered, 2D-nanoparticle based aqueous fluids that can be used as a formulation base for waterborne paints which dry into anisotropic composites. By using largely varying polymer colloids that are negatively charged and thus exert depletion forces on the *α*-ZrP platelets we provide experimental support for recent simulations on the phase behaviour of discotic dispersions with coexistent isotropic-nematic-columnar arrangements of 2D particles. Finally, we emphasize the affordable and innoxious chemistry of the colloidal system as well as the convenient use of a storage stable and readily dispersible intermediate.

## Methods

### Materials and sample preparation

All used materials are listed with CAS numbers, supplier and purity in the supporting information. Synthesis and purification of *α*-ZrP, exfoliation processes with amines and colloidal processing as well as the preparation of samples with polymer colloids are described in detail in the supporting information. Syntheses were performed on decimolar scale while samples for analysis and rheological characterization were prepared in quantities of 10 to 100 ml. For mixing only blade and magnetic stirring was applied if at all. All samples were stored under ambient conditions and not shielded against artificial lighting. Cuvettes for long time observation were additionally sealed with parafilm (Parafilm M, Bemis, Neenah, WI, USA). Samples for film formation studies were drawn manually with a frame (150 *μ*m slit) on Kapton® foil (Kapton 150FN999, DuPont, Midland, MI, USA) under ambient conditions. If not noted otherwise, all volume fractions are calculated by taking into account the amine loss during the centrifugation and the freeze-drying step as well as the amount of physisorbed water in the obtained powder.

### Characterization and testing

X-ray diffraction patterns were recorded on a SAXSess mc^2^ diffractometer (Anton-Paar, Graz, AT) using line collimation and Cu-K*α* radiation.

Small-angle X-ray scattering (SAXS) was carried out on the ID02 high brilliance beamline of ESRF, Grenoble, France, using monochromatic radiation (*λ* = 0.1 nm), a source-sample distance of 55 m and sample-detector distances (max range 1 to 10 m) to cover a typical *q*-range of 0.0025–11 nm^−1^. Intensities were corrected for empty glass capillaries for liquid samples and Kapton foil for drying films.

For SEM-EDX of solid *α*-ZrP, samples were fixed on conductive carbon tape (PLANO Leit-Tabs, Plano, Wetzlar, Germany) and coated with a few nanometers of platinum to enhance the electric conductivity (POLARON SC 7640, Quorum Technologies Ltd, Laughton, UK). Scanning electron microscopy was performed with a HITACHI SU 8230 (Hitachi, Chiyoda, Tokyo, Japan). EDX spectra were recorded with a BRUKER QUANTAX equipped with detectors XFlash 6|30 and FlatQUAD 5060F (Bruker, Billerica, MA, USA).

For cryo-SEM analysis liquid samples were sandwiched between two aluminium platelets, each with a milled depth of 100 *μ*m. High pressure freezing with liquid nitrogen was performed within the HPM 100 apparatus (Leica Microsystems, Wetzlar, Germany). Freeze-fracture was done using the BAF 060 apparatus (Baltic Praeparation, Niesgrau, Germany) and the fracture surfaces were typically freeze-etched for 3–6 min at 173 K and subsequently sputter coated with tungsten, a first layer of 3 nm was deposited under an angle of 45° and a second layer of 3 nm under 90°. For imaging samples were transferred by the shuttle system VCT-100 (Leica) into a Zeiss Leo microscope (Carl Zeiss, Jena, Gaermany). An acceleration voltage of 2 kV was used and secondary electrons were detected by the InLens detector.

Liquid *α*-ZrP-amine samples for TEM analysis were diluted with water to 0.01 weight-% and a 1 $\mu \ell$ droplet was allowed to dry on carbon foil. Dried films on Kapton® foil were frozen in liquid nitrogen and ultrathin sections of 70–120 nm were cut at −80 °C using an UC6 ultra-microtome equipped with an EM FCS cryo chamber and a diamond knife (Leica Microsystems). Cryogenic transmission electron microscopy (cryo-TEM) was performed on a Tecnai G2 electron microscope (FEI, Hillsboro, NJ, USA) using 200 kV accelerating voltage.

Rheological characterization was performed on an Anton-Paar rheometer MCR302 with cone plate CP50-1-SN6017 at 23 °C using a cone plate distance of 0.05 mm at a constant frequency of 1 Hz.

Cross-polarized light microscopy was performed with an Olympus BX51 microscope (Olympus, Shinjuku, Tokyo, Japan) equipped with an UM Plan FI-lens and a XC10 digital camera. A droplet of the sample was deposited on a glass slide and covered with a cover slip under ambient conditions.

Particle size distribution was analyzed via dynamic light scattering using a Malvern Zetasizer Nano S 90 (Malvern Panalytical, Malvern, UK) and polystyrene cuvettes. Typically, concentrations were set to 0.05 weight-%.

Zr- and P-contents were determined by ICP-OES on an Ametek Spectroblue TI (Ametek Inc., Newark, DE, USA). 1 ml of sample solutions was typically diluted with 49 ml of water and 1 ml of nitric acid (65%) was added before measurement. TGA was carried out using a Shimadzu DTG-60 (Shimadzu Corp., Kyoto, Japan) with alumina cell at a heating rate of 10 °C/min in nitrogen atmosphere. Titration for meq base was done with a Metrohm Titrando 808 (Metrohm AG, Herisau, Switzerland).

A Bruker Dimension Icon apparatus was used for AFM measurement using a TESPA probe. Scans were performed at room temperature in air using a peak force frequency of 20 kHz.

The program SASfit (http://sasfit.org/releases) was used for SAXS curve simulations (J. Appl. Cryst. 48, 1587 (2015)). Crystal structures were created by means of the VESTA program (J. Appl. Cryst. 44, 1272 (2011)) and molecular structures were visualized with the open-source Java viewer for chemical structures in 3D, Jmol (http://www.jmol.org/).

## Supplementary information

Supplementary Information
