# Supplementary material for: Hierarchically Ordered α-Zirconium Phosphate Platelets in Aqueous Phase with Empty Liquid
Source: Sci Rep. 2019 Nov 8;9:16389. doi: 10.1038/s41598-019-51934-y (PMC6841702; doi:10.1038/s41598-019-51934-y)
Supplement: Supplementary file 1 — Supplementary Information [file 41598_2019_51934_MOESM1_ESM.pdf]

Hierarchically Ordered α-Zirconium Phosphate Platelets in Aqueous Phase with Empty Liquid

Xi Lin[1], Dirk Schmelter[1], Sadaf Imanian[1], and Horst Hintze-Bruening[1,*]

[1]BASF's Coatings Division, 48165 Muenster, Germany
[*]horst.hintze-bruening@basf.com

Supporting Information

Materials

All chemicals and technical products were used as received.

Deionized water was used for syntheses and formulations.
Zirconium oxychloride $ZrO(Cl)_2 \cdot 8\ H_2O$; CAS [13520-92-8]; Honeywell; $\geq$ 99.5 %
N-Methyldiethanolamine (MDEA); CAS [105-59-9]; Aldrich; $\geq$ 99 %
1-Hydroxyethane-1,1-diphosphonic acid (HEDP); CAS [2809-21-4]; Aldrich; 60 % in H_2O
Tris(2-hydroxymethyl)amine (TEA); CAS [102-71-6]; Sigma-Aldrich; $\geq$ 99.0 %
2-Dimethylaminoethanol (DMEA); CAS [108-01-0]; BASF; $\geq$ 99.6 %
Bis(2-hydroxypropyl)amine (DIPA); CAS [110-97-4]; Sigma-Aldrich; $\geq$ 98.0 %
Tris-(2-hydroxypropyl)amine (TIPA); CAS [122-20-3]; TCI; > 98.0 %
Tris(hydroxymethyl)aminomethane (TRIS); CAS [77-86-1]; Sigma-Aldrich; $\geq$ 99.8 %
Pyrimidine (PYR); CAS [289-95-2]; Sigma-Aldrich; $\geq$ 98.0 %
Sodium chloride (NaCl) [CAS 7647-14-5], Fluka, $\geq$ 99.5 %
Magnesium chloride ($MgCl_2$); CAS [7786-30-3]; Alfa Aesar; 99.99 %
Sodium sulfate (Na_2SO_4); CAS [7757-82-6]; Aldrich; > 99 %
Polymer dispersions PES and PUR_a have been described in detail as PES_h / PES_{NB} and PUR_h in preceding articles.[1,2]
PUR-PAC is the commercial product DAOTAN®TW 6464 36WA from Allnex B. V. (Bergen op Zoom, Netherlands).
PUR_b, PUR-μ-gel and PAC-μ-gel are proprietary, non-commercial polymer dispersions of BASF Coatings Division.

Physical and Chemical Data of Hydroxyalkyl Amines

Boiling points and solubilities are from technical data sheets or material safety data sheets released by the supplier (for TRIS: Merck) or the manufacturer (Dow Chemical Company), respectively.

The pK_a values have been reported by Hamborg and Versteeg[3] (for TEA, MDEA, DIPA and DMEA), by Subirats et al.[4] as well as Beckers et al.[5] (8.08 vs. 8.24 for TRIS). The pK_a values of TIPA was taken from the material safety data sheet (Sigma-Aldrich).

Sample Preparations

α-ZrP = $Zr(HPO_4)_2 \cdot H_2O$

20 g of $ZrO(Cl)_2 \cdot 8H_2O$ were refluxed in 200 ml of 6 M phosphoric acid at 100 °C, with a stirring speed of 400 rpm and under nitrogen protection. After 24 hours reaction, α-ZrP was collected by centrifugation for 30 minutes. To get rid of H_3PO_4 and HCl, α-ZrP was centrifuged at 16,233 x g for 60 minutes and the sediment redispersed in water (the centrifuge tubes with the sediment are filled with water which results in a roughly 10fold dilution) and centrifuged at 16,233 x g for 10 minutes at least 4 times or until $AgNO_3$ addition didn't cause any haze from AgCl. For the test 3 drops of a roguhly 2% solution of silver nitrate in dilute nitric acid (pH 1-2) were added to 3-4 ml of the sample solution. The product was dried at 65 °C for 12 hours under air.

α-ZrP-MDEA, Standard Process

43.39 g α-ZrP were dispersed in 600 g deionized water and stirred with a dissolver at 1,800 rpm for 15 minutes to obtain 6.7 weight-% α-ZrP (0.24 mol/l). The suspension was transferred into an 1,000 ml 3-neck round-bottom flask and 34.32 g

methyldiethanol amine (0.48 mol/l) were added at once under magnetic stirring and ambient conditions. The dispersion was stirred at 1,200 rpm for 24 hours. The product was fractionated by centrifugation at 16,233 x g for 2 h. The supernatant was discarded and the gel-phase was freeze-dried using a Christ Epsilon 2-4 LSC freeze drier (Christ GmbH, Osterode, Germany). The sample was frozen at -85 °C before evacuation. For the sublimation phase the pressure was set to 50 Pa with shelf and ice-condenser temperatures of +20 °C and -20 °C respectively. For the final drying/desorption the shelf temperature was raised to 40 °C and the pressure lowered to 1-10 Pa for approximately 1 h.

The same procedure was used with the amines TEA, DMEA, DIPA, TRIS and PYR. The latter amine was additionally used in tenfold excess relative to HO-P in α-ZrP.

α-ZrP-MDEA, Exfoliation at 273 K

The same protocol as described for the standard process was observed with the following adaptations. The α-ZrP dispersion was transferred to a double-walled glass-reactor and cooled to 0°C with a Lauda RK8 KP cryostat (Lauda GmbH, Lauda-Koenigshofen, Germany) under stirring with a blade agitator at 850 rpm. Cooling was maintained until the addition of MDEA was accomplished. The amine was added dropwise over 70 minutes and the stirring speed was gradually increased to 2,000 rpm in order to avoid the formation of an organic top layer of the amine. The temperature of the reaction mixture increased up to 5.7°C over the addition period. After MDEA addition the batch was stirred at 1,200 rpm under ambient conditions for 24 h.

Tangential Flow Fractionation

Tangential flow filtration was performed with a Cogent μScale TFF system (Millipore, Burlington, MA, USA) equipped with Pellicon XL modules of different pore sizes and membrane materials. To achieve processability the samples were diluted twentyfold with water. Depending on the specifics of the respective sample, pump speed and retentate pressure were adjusted to facilitate cross-membrane flow while keeping the feed pressure below 4.5 bar and assuring a maximum trans-membrane pressure of 2.5 bar. The retentate was recirculated and the lacking volume in the reservoir was replaced with water and the pH, which typically was in the range of 8 - 9, was adjusted with a 1 weight-% solution of the amine to the pH value of the starting mixture. The process was repeated five times for a total permeate volume of at least 800 ml. Afterwards the membrane was subsequently flushed with water, 0.1 M NaOH and finally water.

α-ZrP-MDEA and Polymer Dispersions

Freeze-dried α-ZrP-MDEA was swollen with at least the same weight of deionized water over night at rest under ambient conditons. Depending on the NVC of the polymer dispersion and the desired weight fractions of α-ZrP-MDEA and solid polymer, stock solutions of α-ZrP-MDEA were prepared by adding water to the swollen α-ZrP-MDEA paste, typically 20 weight% and 40 weight-% in amounts of 10 g. Appropriate amounts of the intended polymer dispersion was added. For all steps shaking of the vial or stirring with a spatula was sufficient.

Table 1. Amine retention in the freeze-dried powder from the centrifuged gel of ten batches of α-ZrP-MDEA was determined for methodology validation. Thus, the products from syntheses with different parameters consisted of particles with different mean particle sizes: $D_z = 150 - 300\,\text{nm}$ from DLS. No impact of the particle size on the amine recovery was observed. The table lists the percentage of MDEA loss with regard to the theoretical content in $[Zr(PO_4)_2 \cdot 2\,\text{MDEA-H}]$. The retrieved MDEA in the freeze-dried powder was determined by means of elementary analysis of carbon and nitrogen as well as the weight loss in thermogravimetric analysis (TGA). Finally, the supernatants of aqueous dispersions of the redispersed powders were titrated for basic compounds. Arithmetic mean values and standard deviations are given.

Method	$\bar{x}$	s
meq base	11.5	5.4
C content	25.0	2.3
N content	26.4	2.6
TGA	22.8	2.2

Roughly half of the amine loss was found with the titration for basic compounds with HCl in the supernatants which supports our observation that repeated fractionation cycles lead to a loss of dispersibility of the freeze-dried product. This suggests distribution equilibria between intercalated MDEA-H^+ and free MDEA in the surrounding liquid.

For the calculation of the MDEA retention from elementary analysis data, the amount of physisorbed water was accounted for. This was attributed to the weight loss in TGA below 80°C which varied over the batches in the range of 3.0 - 4.2 weight-%. The solid residue at 800°C was attributed to ZrP_2O_7 ($M = 265.17$) which was used to calculate the actual MDEA content in the "solid" fraction of the freeze-dried powder above 80°C. A typical TGA curve is shown in figure SI11.

fraction	$m_{permeate}$ [g]	Zr [ppm]	P [ppm]	Na [ppm]	Zr [%]	P [%]	P/Zr (equiv.)
1	173	7	55	0	0.0715	0.8263	23
2	74	5	45	0	0.0218	0.2892	27
3	201	3	38	0	0.0357	0.6666	37
4	186	3	30	0	0.0330	0.4865	29
5	174	2	27	0	0.0206	0.4092	40
6	200	110	85	0	1.2991	1.4782	2
7	203	1	6	2,300	0.0120	0.1062	18
8	199	1	1	65	0.0118	0.0174	3

Table 2. Typical TFF protocol of the purification of a diluted α-ZrP-MDEA gel phase *"273"* (exfoliated at 0°C) over a Biomax® membrane (5 kDa). ICP-OES results for Zr, P and Na content in the permeates are given in ppm. The results were converted into percentages of the respective contents in the starting material and the P/Zr equivalent ratios were calculated. The permeate fractions 1 - 5 are from the purification process, with the last (5) being the first testing negative for Cl^-. The membrane was subsequently flushed with water (fraction 6), 0.1 M NaOH (fraction 7) and water (fraction 8). The table lists the Na, P and Zr contents of the washing solutions.

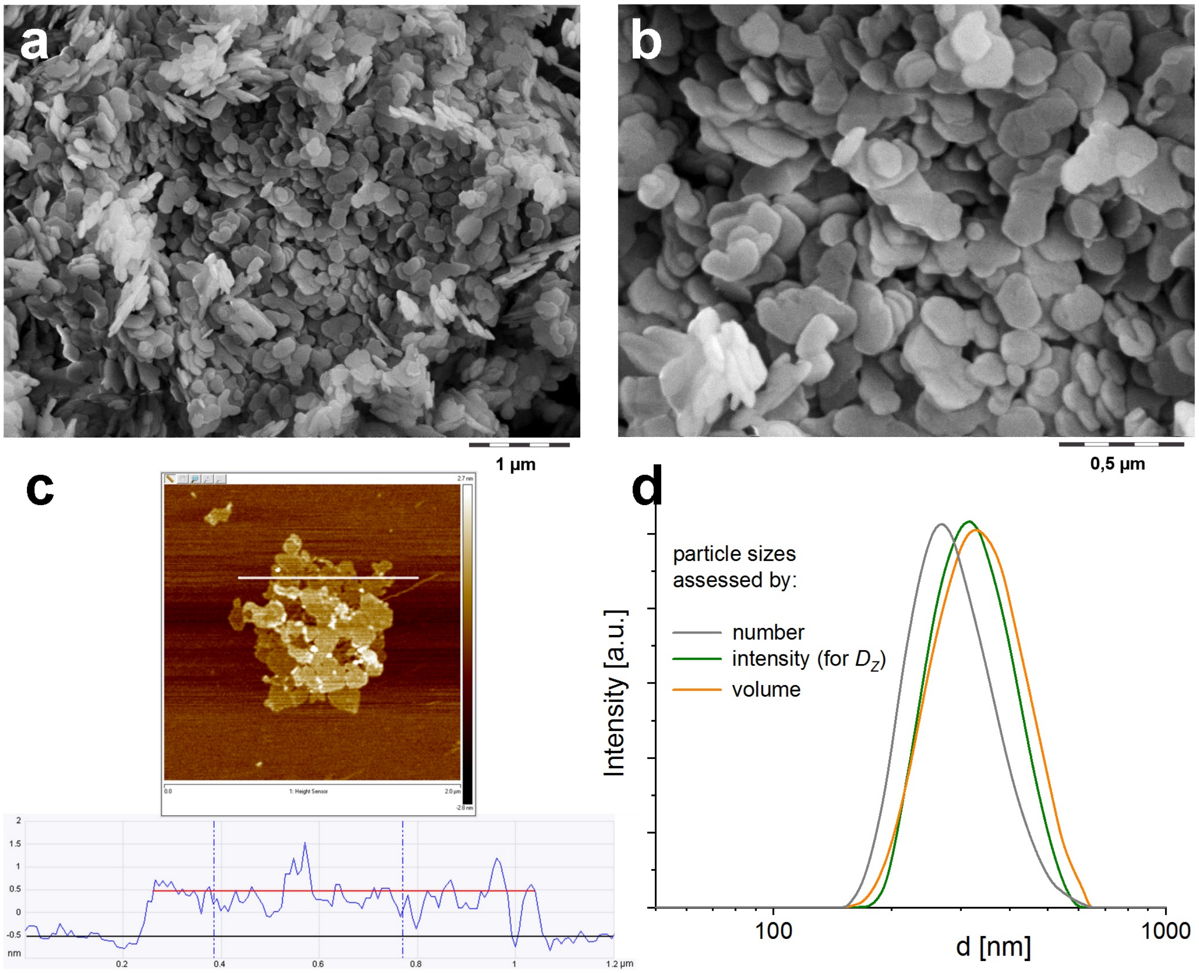

Figure 1. SEM pictures of pristine α-ZrP with increasing magnification of 20 k (a), 50 k (b) (b). (c) AFM topography of α-ZrP-MDEA platelets, deposited on mica from aqueous dispersion ($w = 0.005$) and rinsed with water with height profile along the white line (image size 4 μm^2). The black and the red line for the substrate and the mean layer height respectively are drawn as a guidance for the eyes. Leaving out spikes from folds or hydrolysis products and valleys from lateral gaps or lacunae, the height of the deposited α-ZrP-MDEA single layer can be estimated to be $\approx$ 1 nm. This value corresponds to the layer thickness ($t = 0.68$ nm) plus one MDEA-H$^+$ layer beneath α-ZrP. Note that the rinsing with water pushed the outward ion pairs Zr-P-O$^-$/$^+$H-MDEA to equilibrate towards P-OH and MDEA, the latter being washed off. The gallery height (h) in restacked α-ZrP-MDEA with a bilayer MDEA-H$^+$ accommodation is $h = d - t = 1.44 - 0.68 = 0.76$ nm (cf. figure SI4, table 1 of article). This is in reasonable accordance with a monolayer MDEA-H$^+$ between mica and α-ZrP ($h/2 \approx 0.36$ nm). (d) Particle size distribution from dynamic light scattering of the diluted gel-fraction of α-ZrP-MDEA, obtained from the standard exfoliation process.

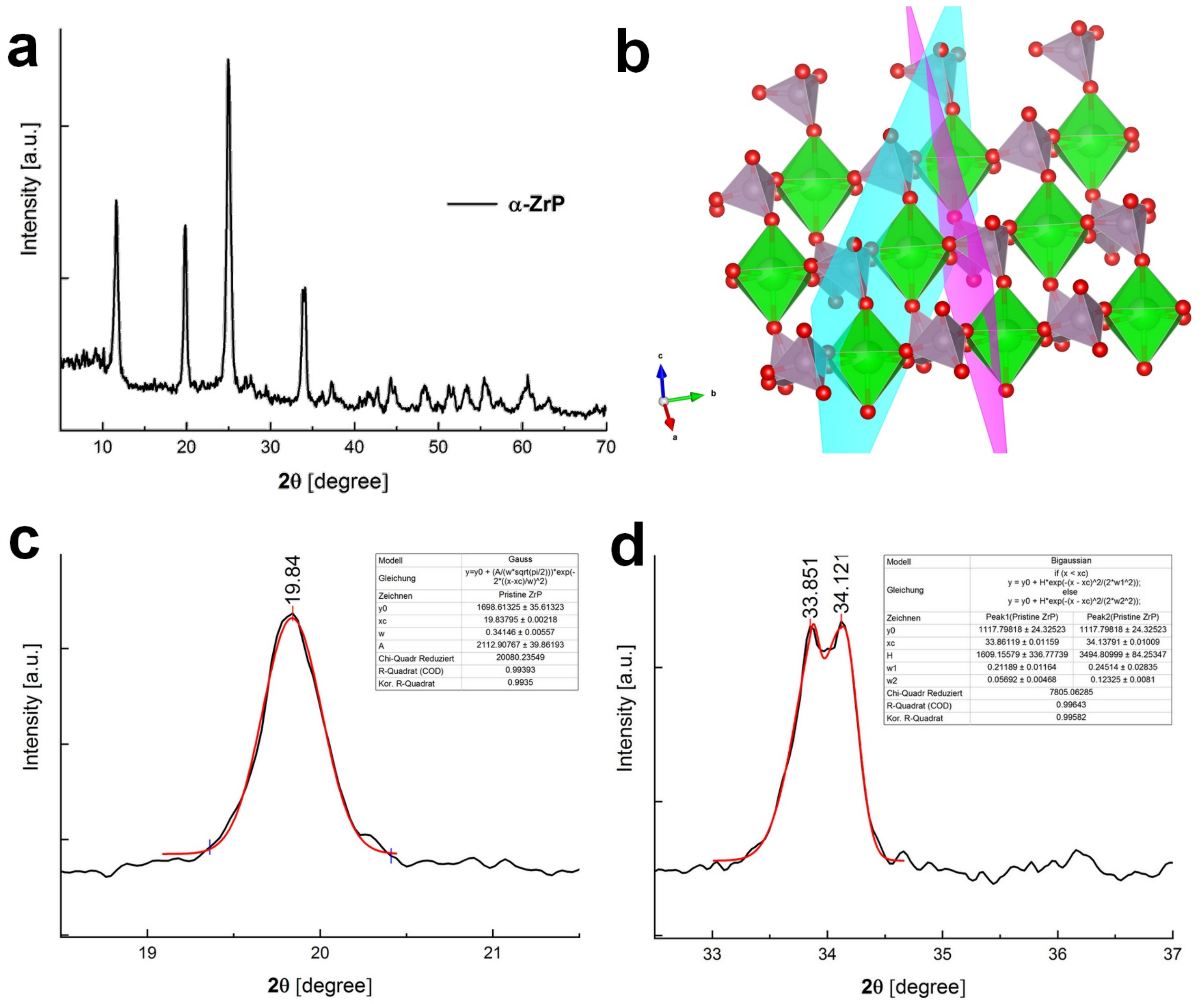

Figure 2. (a) XRD curve for α-ZrP. (b) Visualization of lattice planes *110* (blue) and *020* (pink) of an α-ZrP layer. (c) Peak for *110* at $2\theta = 19.84$ (19.8 calculated) and curve fit (Gaussian, red line). (d) Peak for *020* at $2\theta = 33.85$ (33.81 calculated) next to the peak for *31(-2)* at $2\theta = 34.12$ (34.10 calculated) and curve fit (bi-Gaussian, red line).

The crystal domain size L in α-ZrP can be estimated using the full width half maximum (FWHM) XRD peak analysis according to Scherrer[6]

$$L = \frac{K \cdot \lambda}{\beta \cdot cos\theta} \tag{1}$$

with the X-ray wave length λ (1.5418 Å), the FWHM β in radian, the cosine of the Bragg angle θ and a dimensionless constant $(0.62 < K > 2.08)$ which depends on the shape of the crystallite and the diffraction plane $(hk\ell)$. Mostly $K = 1$ is used as an approximate.[7] For the estimation of β of the peaks at $2\theta = 19.84$ and $2\theta = 33.85$ these are simulated using the Gaussian and bi-Gaussian model,[8] respectively (vide infra, cf. fig. 2c,d). For the *110* lattice plane $(w = 0.342)$ a FWHM of $\beta = 0.007$ yields a domain size $L = 22$ nm. In the *020* plane a peak width of $(w_1 + w_2 = 0.212 + 0.057 = 0.27)$ is considered for $\beta = 0.0047$ and a domain size of $L = 34$ nm. In the light of these approximations we conclude a practically relevant domain size in the order of 25 - 30 nm.

Corresponding peak analyses for restacked α-ZrP-MDEA are hampered by poor data quality in conjunction with more overlapping peaks around $2\theta = 34$. However, a rough estimation yields the same order of magnitude for the intra-layer long-range order.

Gaussian fit

$$y = y_0 + \frac{A}{w\sqrt{\pi/2}} e^{-2\frac{(x-x_c)^2}{w^2}} \tag{2}$$

FWHM= $w\sqrt{ln4}$ ($w = 0.342^\circ$)

Bi-Gaussian fit using Hermite polynomials (*He*)

$$y = y_0 + He^{-0.5(\frac{x-x_c}{w_1})^2} for x \leq x_c \qquad y = y_0 + He^{-0.5(\frac{x-x_c}{w_2})^2} for x \geq x_c \tag{3}$$

Peak 1 ($2\theta = 33.85$) $w_1 + w_2 = 0.212 + 0.057 = 0.27^\circ$
Peak 2 ($2\theta = 34.12$) $w_1 + w_2 = 0.245 + 0.123 = 0.37^\circ$

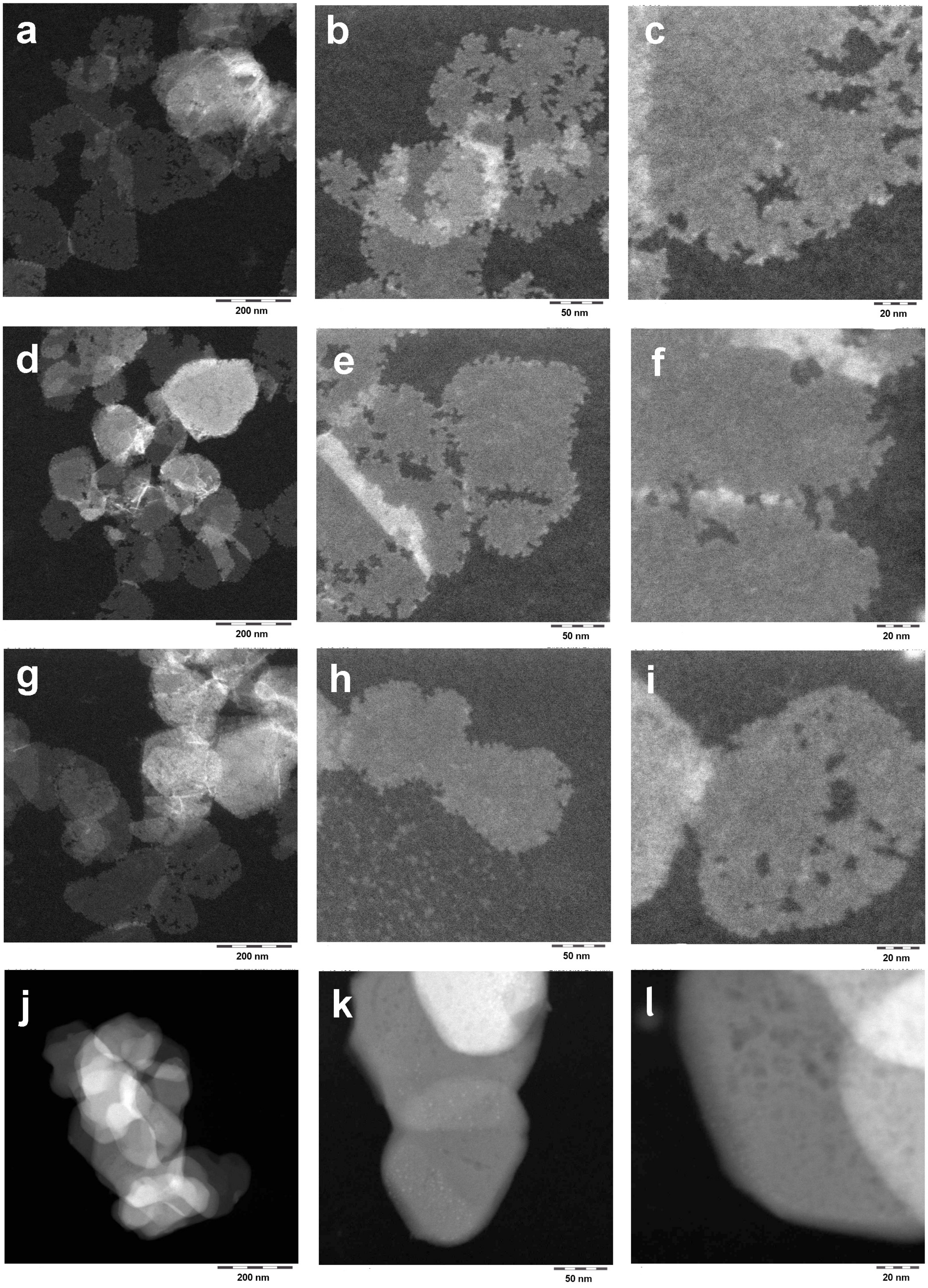

Figure 3. HAADF-TEM images of residues from diluted α-ZrP-amine gels with increasing magnification from left to right: (a-c) DIPA, (d-f) TIPA, (g-i) TRIS and (j-l) excessive PYR.

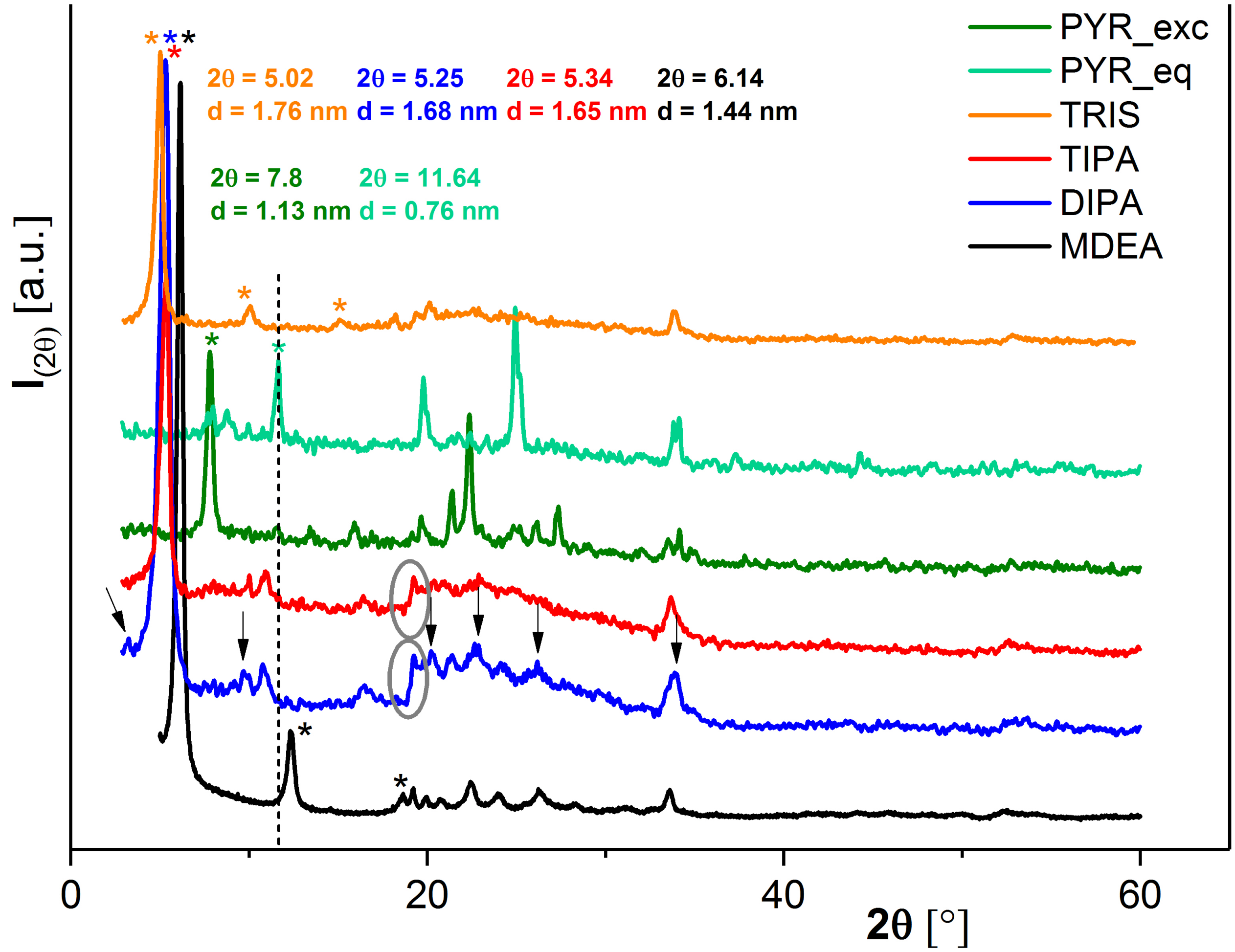

Figure 4. Vertically shifted powder XRD recordings of α-ZrP-amine products, obtained from the standard exfoliation process. Asterisks indicate harmonic peaks for the stacked α-ZrP layers and corresponding repeat distances obtained from the 1st (harmonic) peaks are listed. The vertical dashed line marks the position of the *002* reflex for pristine α-ZrP stacking which is only visible in the curve from α-ZrP-PYR$_{eq}$. Note the distinct sharpness and intensity of peaks from lattice plains involving interlayer distances in α-ZrP-PYR$_{exc}$. These indicate a high stacking order that is presumably induced by homogeneously aligned PYR fitting the triangular lacuna corned by apical P-OH groups of α-ZrP layers. On the contrary, encircled asymmetric peaks from α-ZrP-DIPA(TIPA) indicate turbostratic disorder in the piling of layers. These may be caused by interstitial species that also give rise to additional peaks which are highlighted by arrows for α-ZrP-DIPA. Interestingly, their positions don't match those in the curve for TIPA. Remarkably, one reflex at $2\theta = 3.24\,°$ may be assigned to a second population with a larger gallery height of 2.72 nm. The occurence of such stacking is backed up by SAXS measurement of the colloidal state (cf. figure SI5).

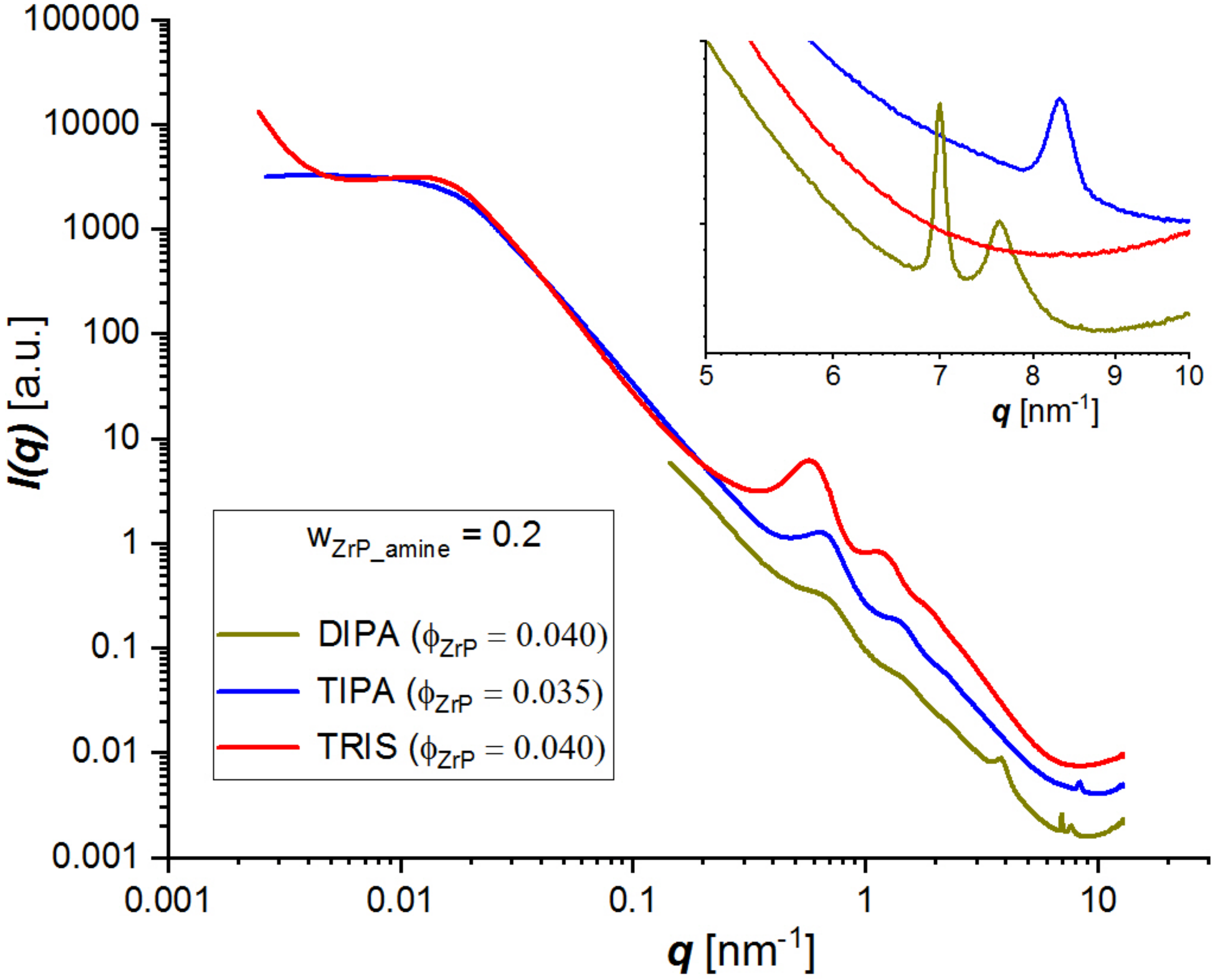

Figure 5. Vertically shifted SAXS recordings of the colloidal states of α-ZrP-amine comprising TRIS, DIPA and TIPA, respectively. Besides the stacking of α-ZrP layers that cause similar humps as seen with α-ZrP-MDEA (cf. fig. 4a in the article), TIPA and in particular DIPA derived dispersions display distinct peaks at large q-values (cf. magnified section in the insert). The peak at $q = 7.6\,\mathrm{nm}^{-1}$ is the second harmonic of the one at $q = 3.8\,\mathrm{nm}^{-1}$ in the main diagram which corresponds to objects or distances of the size of $d = 1.65\,\mathrm{nm}$ whereas the sharp peak at $q = 6.99\,\mathrm{nm}^{-1}$ reflects another distance or object with $d = 0.9\,\mathrm{nm}$. We note that these peaks can not be assigned to those of pristine α-ZrP. A plausible scenario would be that a second phase consists of DIPA intercalated though not exfoliated layers (compare to the main peak in XRD, fig. SI4), whereas Zr-clusters ($d = 0.9\,\mathrm{nm}$) are populating galleries of exfoliated layers. During drying these clusters stay in the interstitials and form a second population in the solid material with a repeat distance of $d = 2.72\,\mathrm{nm}$ according to a peak at $2\theta = 3.24\,°$ (cf. fig. SI4). No information can be given about the spatial distribution of such a stacking: intra-particle domains with different repeat distances versus coexisting particles with different but uniform stackings.

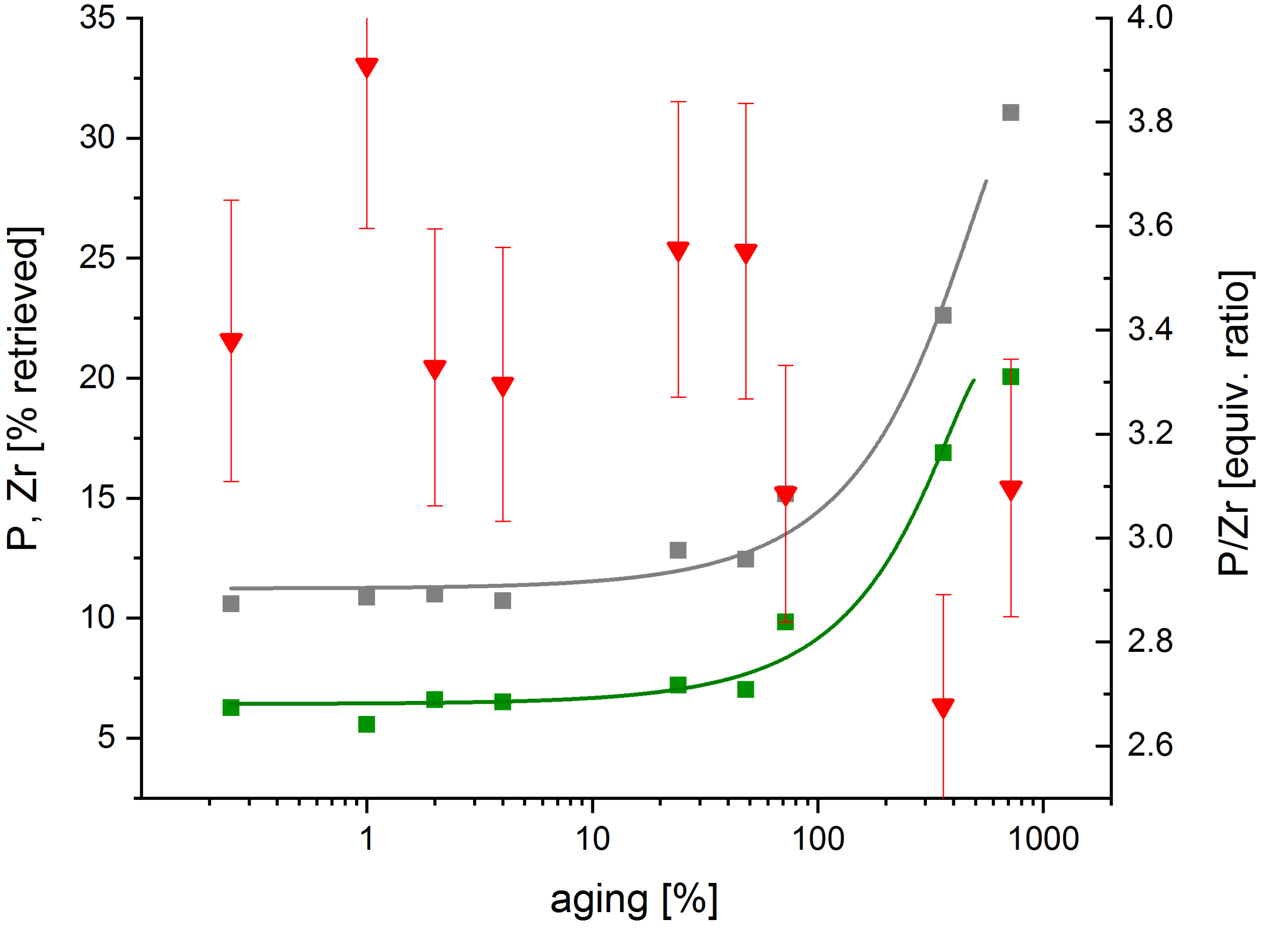

Figure 6. ICP-OES results from the supernatants of centrifuged samples taken with progressing time from an aging batch of MDEA exfoliated α-ZrP which was stirred magnetically under ambient conditions. Data are expressed as retrieved fractions of the initial amount of α-ZrP for analyzed phosphorous (grey) and zirconium (green) in percent (lines are just a guidance for the eyes). Red triangles depict the atomic equivalent ratios of P:Zr that are found in the supernatants. The data unambiguously show that phosphorous is preferentially detected in contrast to a P:Zr-ratio of two that is found for a non centrifuged control sample (not shown), the latter being in accordance with the chemical formula of α-ZrP. Despite large experimental error a trend to lower ratios is seen over the entire time frame.

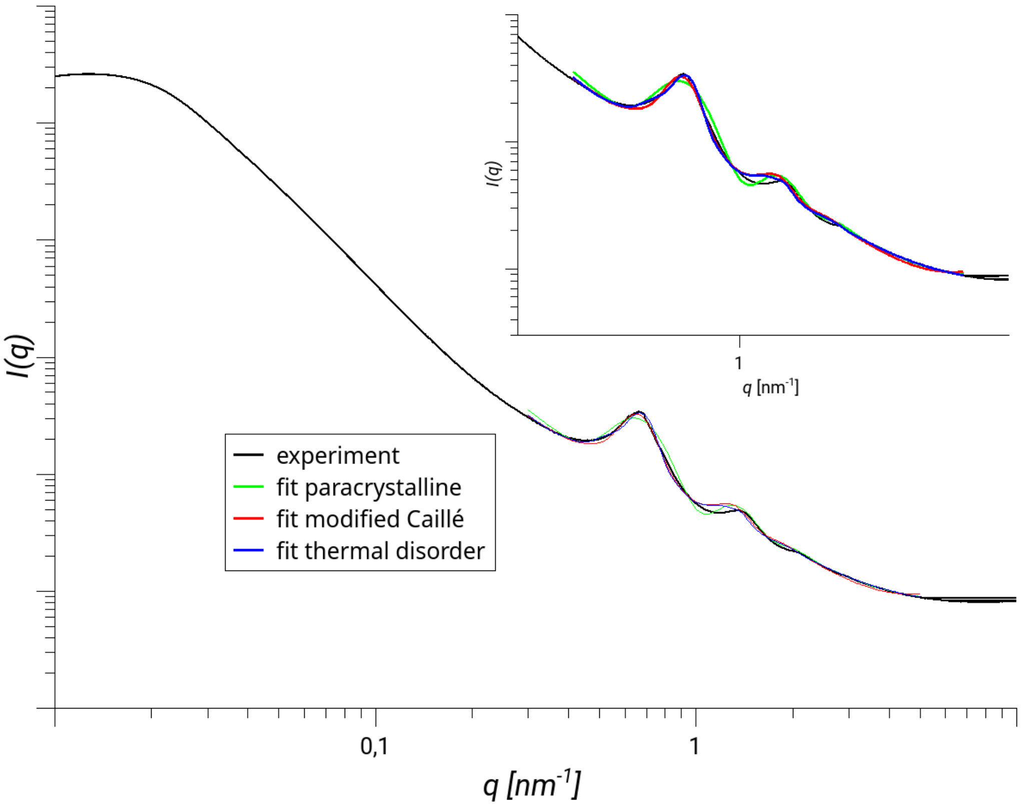

Figure 7. Simulations of the SAXS q-range from $0.3\,\mathrm{nm}^{-1}$ to $5\,\mathrm{nm}^{-1}$ for the scattering from an α-ZrP-MDEA dispersion ($w = 0.2$). Homogeneous, monodisperse platelets are assumed ($t = 0.68\,\mathrm{nm}$, $L = 300\,\mathrm{nm}$). For the structure factor either the paracrystalline model, the modified Caillé theory or the thermal disorder algorithm have been used. In all cases the experimental curve could be reasonably approximated assuming few layers in the scattering domain ($N = 3-4$) and parameters for the bending of layers (η) and distance fluctuations (δ) respectively. Especially for large q-values background scattering is added in order to fit the curve. Fits were performed using the program SASfit, version 0.94.11.[9]

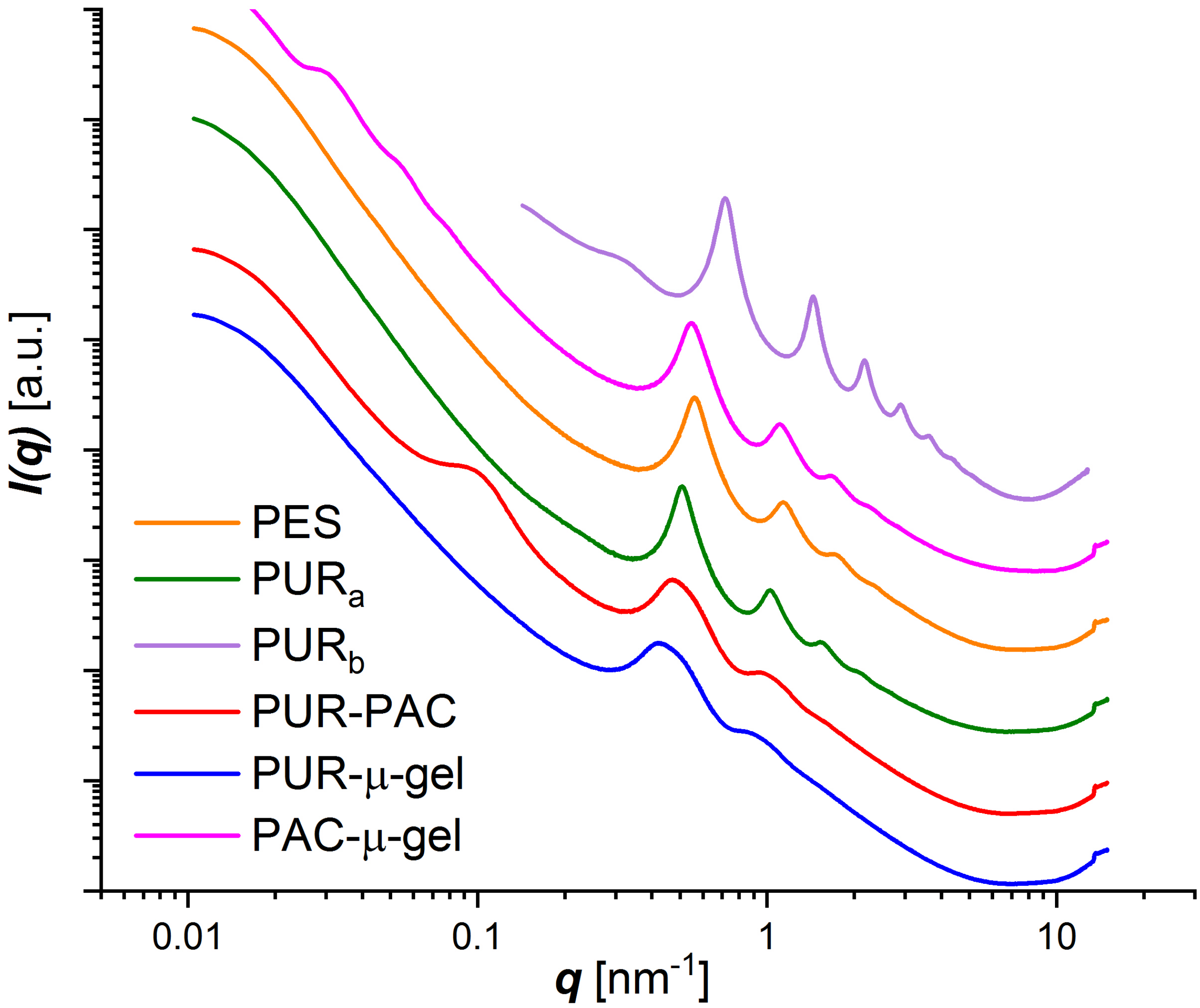

Figure 8. Vertically shifted SAXS curves from dispersions of α-ZrP-MDEA ($w = 0.1$) with polymer colloids ($w = 0.1$). Repeat distances for stacked α-ZrP layers are smaller than in the corresponding neat α-ZrP-MDEA dispersion ($d = 15.7$ nm): 8.85 nm (PUR_b), 11.2 nm (PES), 11.4 nm (PAC-μ-gel), 12.3 nm (PUR_a), 13.1 nm (PUR-PAC), 14.3 nm (PUR-μ-gel). Note that the scattering curves of dispersions with PAC-μ-gel, PUR-PAC and PUR_b show humps caused by the polymer colloids. Their sizes, caused by electron density jumps, may differ from the hydrodynamic diameter seen with DLS (table 4 of the article).

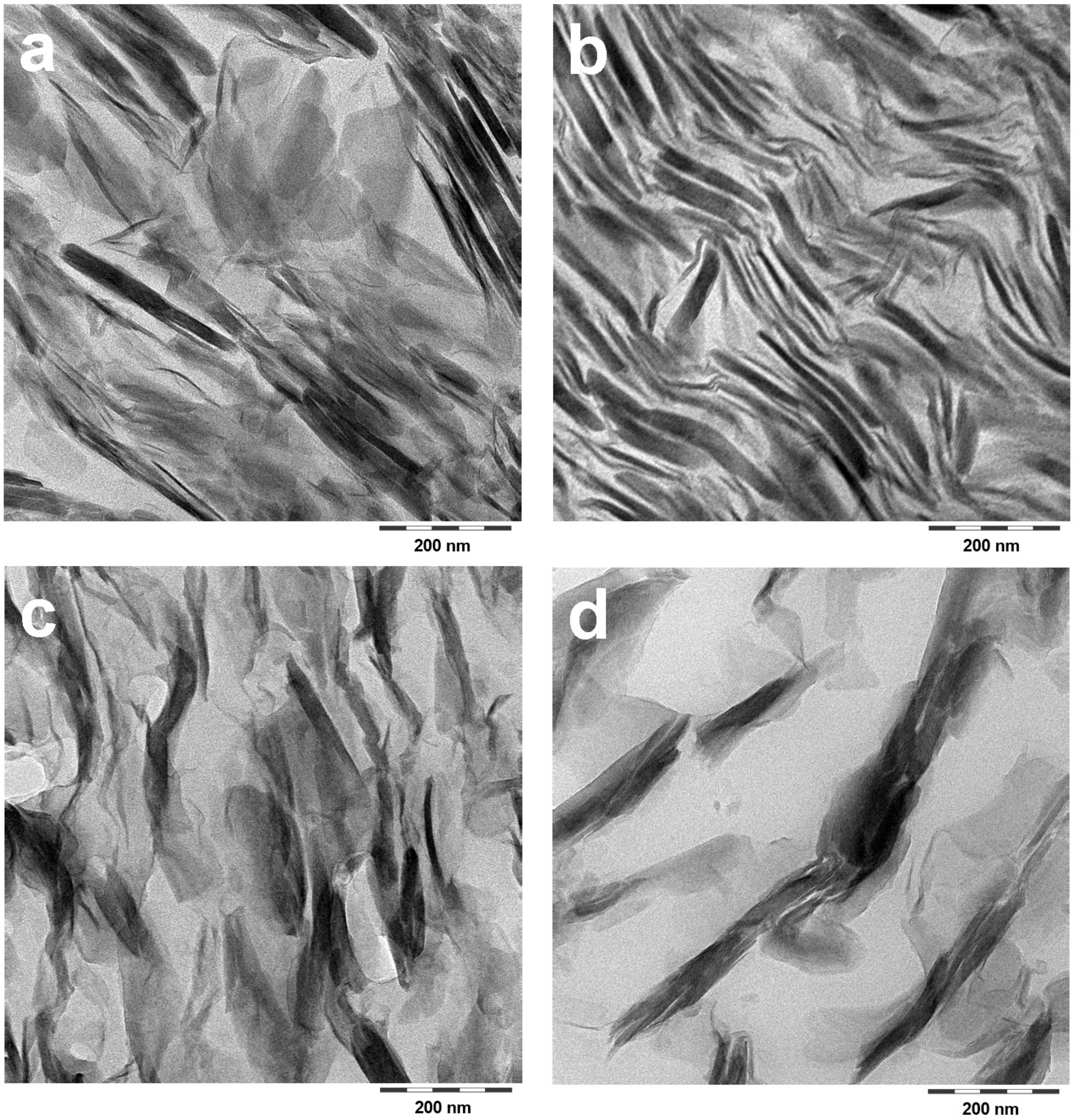

Figure 9. Dried films from α-ZrP-MDEA-polymer dispersions that were drawn on Kapton® foil for real-time SAXS monitoring of the film drying. Cryo-microtomed cross sections are imaged in brightfield TEM analysis. All films comprise equal weight proportions of α-ZrP-MDEA and polymer, respectively. Polymers are (a) PUR_a, (b) PES, (c) PUR-PAC and (d) PAC-μ-gel. Scale bars are 200 nm.

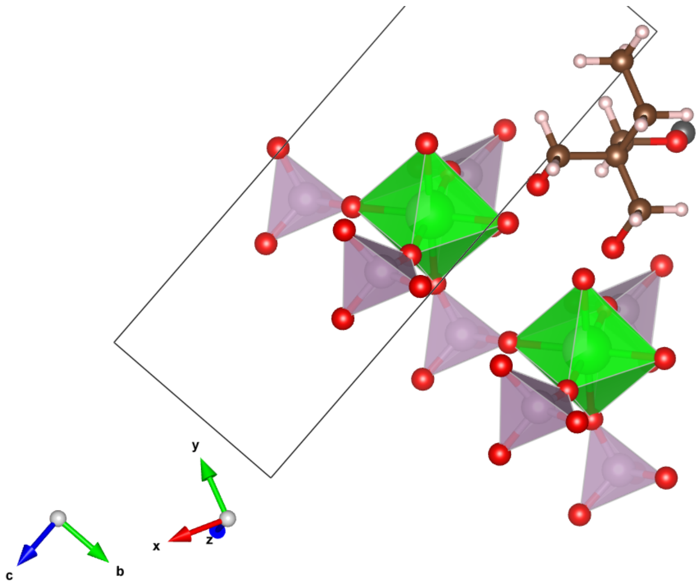

Figure 10. Sketch illustrating the molecular dimensions of adjacent Zr-sites at the rim of an α-ZrP layer in relation to geminal hydroxyl ligands. The latter are from trimethylolpropane mono ester end groups of PUR_a polymer chains (symbolized as dark grey sphere pointing to the background). We postulate bidentate liganding to two neighboring Zr-sites as a plausible explanation for the exceptionally high viscosity increase observed with combined α-ZrP-MDEA / PUR_a dispersions.

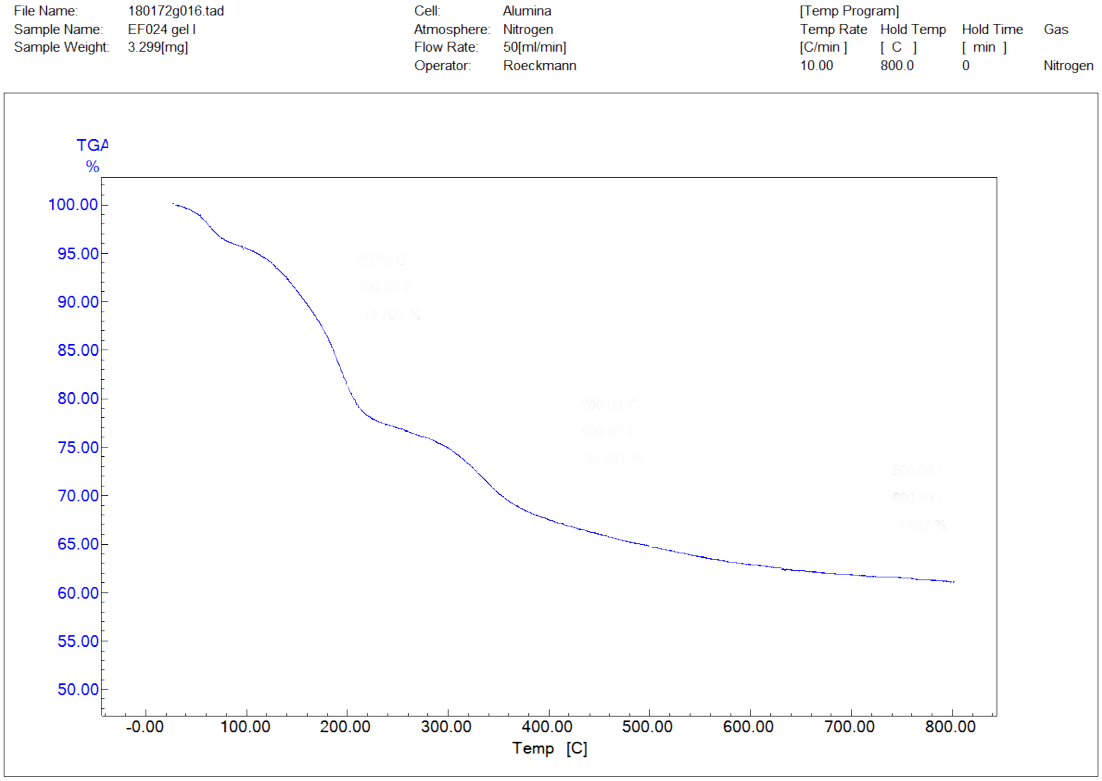

Figure 11. TGA curve of a freeze-dried gel phase of α-ZrP-MDEA (cf. table 1 of SI).

References

1. Troutier-Thuilliez, A., Hintze-Bruening, H., Taviot-Gueho, C., Verney, V. & Leroux, F. Exfoliation and liquid crystal phase formation of layered double hydroxide into waterborne polyurethane coatings. *Soft Matter* **7**, 4242–4251 (2011).
2. Lohmeier, T., Bredol, M., Schreiner, E. & Hintze-Bruening, H. Ordered liquids and hydrogels from alkenyl succinic ester terminated bola-amphiphiles for large-scale applications. *Soft Matter* **10**, 6237–6248 (2014).
3. Hamborg, E. & Versteeg, G. Dissociation constants and thermodynamic properties of amines and alkanolamines from (293 to 353) k. *J. Chem. Eng. Data* **54**, 1318–1328 (2009).
4. Subirats, X., Bosch, E. & Rosés, M. Retention of ionisable compounds on high-performance liquid chromatography XIX. pH variation in mobile phases containing formic acid, piperazine and TRIS as buffering systems and methanol as organic modifier. *J. Chromatogr. A* **1216**, 5445–5448 (2009).
5. Beckers, J., Ackermans, M. & Boček, P. Capillary zone electrophoresis in methanol: Migration behavior and background electrolytes. *Electrophoresis* **24**, 1544–1552 (2003).
6. Scherrer, P. Bestimmung der Groesse und der inneren Struktur von Kolloidteilchen mittels Roentgenstrahlen. *Nachr. Ges. Wiss. Goettingen* 98–100 (1918).
7. Langford, J. & Wilson, A. Scherrer after sixty years: A survey and some new results in the determination of crystallite size. *J. Appl. Cryst.* **11**, 102–113 (1978).
8. Yu, T. & Peng, H. Quantification and deconvolution of asymmetric LC-MS peaks using the bi-Gaussian mixture model and statistical model selection. *BMC Bioinforma.* **11**, 559 (2010).
9. Bressler, I., Kohlbrecher, J. & Thunemann, A. SASfit: a tool for small-angle scattering data analysis using a library of analytical expressions. *J. Appl. Cryst.* **48**, 1587–1598 (2015).
